# The social and economic burden of retinopathy of prematurity: screening, treatment, and health policy perspectives in a transnational narrative review

**DOI:** 10.3389/fped.2026.1894390

**Published:** 2026-08-26

**Authors:** Maria Ester Canepa, Massimiliano Serafino, Pasquale Striano, Luca A. Ramenghi

**Affiliations:** 1 Department of Neuroscience, Rehabilitation, Ophthalmology, Genetics and Mother-Child Health, University of Genoa, Genoa, Italy; 2 IRCCS Istituto Giannina Gaslini, Genoa, Italy

**Keywords:** blindness, neonate, policy, ROP, screening

## Abstract

Retinopathy of prematurity (ROP) is a leading preventable cause of childhood blindness, particularly among premature infants. This transnational narrative review synthesizes international evidence on the economic and social burden of ROP from 2003 to 2025, focusing on the cost-effectiveness of screening, diagnostic imaging, and treatment strategies. A structured PubMed search using the terms “ROP neonate social cost” and “economic impact of ROP” was supplemented through the “Similar Articles” and “Cited Articles” functions. The review included cost-effectiveness analyses, cost-utility studies, burden-of-disease reports, and policy-related evidence published in all languages. Following the thematic framework proposed by Rivero-Arias et al. (2024), the evidence was synthesized to explore the economic implications of ROP screening and management while informing future policy directions. Evidence from 15 eligible studies identified through the primary search was complemented by 71 additional relevant publications identified through PubMed citation tracking. The available evidence suggests that AI-assisted telescreening and smartphone-based imaging may represent cost-effective and scalable screening strategies, while mobile screening units and national telemedicine networks appear feasible in low- and middle-income countries. The reviewed literature also indicates that the long-term societal costs associated with ROP-related blindness substantially exceed those of timely screening and early intervention. However, important evidence gaps remain regarding long-term follow-up costs, the comparative cost-effectiveness of anti-vascular endothelial growth factor therapy vs. laser photocoagulation, and the economic consequences of delayed or missed screening. Successful ROP care models involve multisectoral collaboration among healthcare systems, education, and technology stakeholders. National initiatives in Argentina, India, Rwanda, and Saudi Arabia illustrate how integrated screening programmes, digital innovation, and international collaboration may reduce preventable ROP-related blindness while improving equitable access to care. From a policy perspective, healthcare systems should consider prioritizing screening programmes, validated telemedicine and AI-assisted technologies, national screening registries, and workforce training through tele-education. Future research should expand societal cost and cost-effectiveness analyses, particularly in high-income countries, strengthen AI validation studies, and develop country-specific economic models reflecting local healthcare resources and neonatal intensive care unit organization. Adaptive screening policies, supported by interdisciplinary collaboration and regular updates of screening guidelines, will be essential to reducing the long-term social and economic burden of ROP.

## Introduction

Within the eye-brain axis's role in visual dysfunctions (1), retinopathy of prematurity (ROP) is a common eye disease affecting preterm neonates that leads to preventable blindness, poor neurodevelopmental outcomes and other ocular comorbidities requiring long-term follow-up (2–5). Additionally, it could be a frequent morbidity (6), even with intraventricular hemorrhage (IVH) (6). The justification of this article relies on the growing epidemiological data on ROP as well as on cost-effectiveness analysis (CEA), social cost studies, etc.). Specifically, this paper aims to summarize the most meaningful and transnational evidence.

From 1985 to 2021, ROP was identified worldwide as a common cause of morbidity in premature infants (7). Although timely identification strategies for high-risk patients are necessary in low- and middle-income countries, high-income countries have the highest prevalence of severe ROP (7) despite the highest concentrations of ROP publications (8) between 1950 and 2020. Notably, “the costs of screening for and treating ROP are small compared with the societal costs of resulting blindness. However, little evidence is available for predicting the effects of changes in patient population, screening schedule or ROP treatments” (9). Compared to the limited economic literature on other childhood blinding disorders, the body of work on ROP is relatively more robust but still incomplete (10). However, “a clearer and more coherent overview is needed to support collaboration between health economists, policy makers, neonatologists, and pediatric ophthalmologists” (11). Unlike previous reviews focusing primarily on costs or individual interventions, the present narrative review integrates social burden, health economics, screening strategies, AI, telemedicine, and policy perspectives across multiple healthcare settings.

### Methodology

On 11th December 2025, papers were identified on PubMed using the search strings “ROP neonate social cost” (most anciently indexed year-2026) and “economic impact of ROP” (most anciently indexed year-2026) from the most ancient, indexed year to the most recent indexed year. Given the narrative nature of this review, PubMed was selected as the primary database because it provides comprehensive coverage of biomedical literature relevant to ROP. We recognized this aspect and the following as a potential limitation in the dedicated section. Moreover, the period 2003–2025 was included to capture contemporary evidence generated after the widespread implementation of modern ROP screening programs and evolving treatment strategies, including digital imaging and anti-vascular endothelial growth factor (VEGF) therapies.

**Figure 1:**
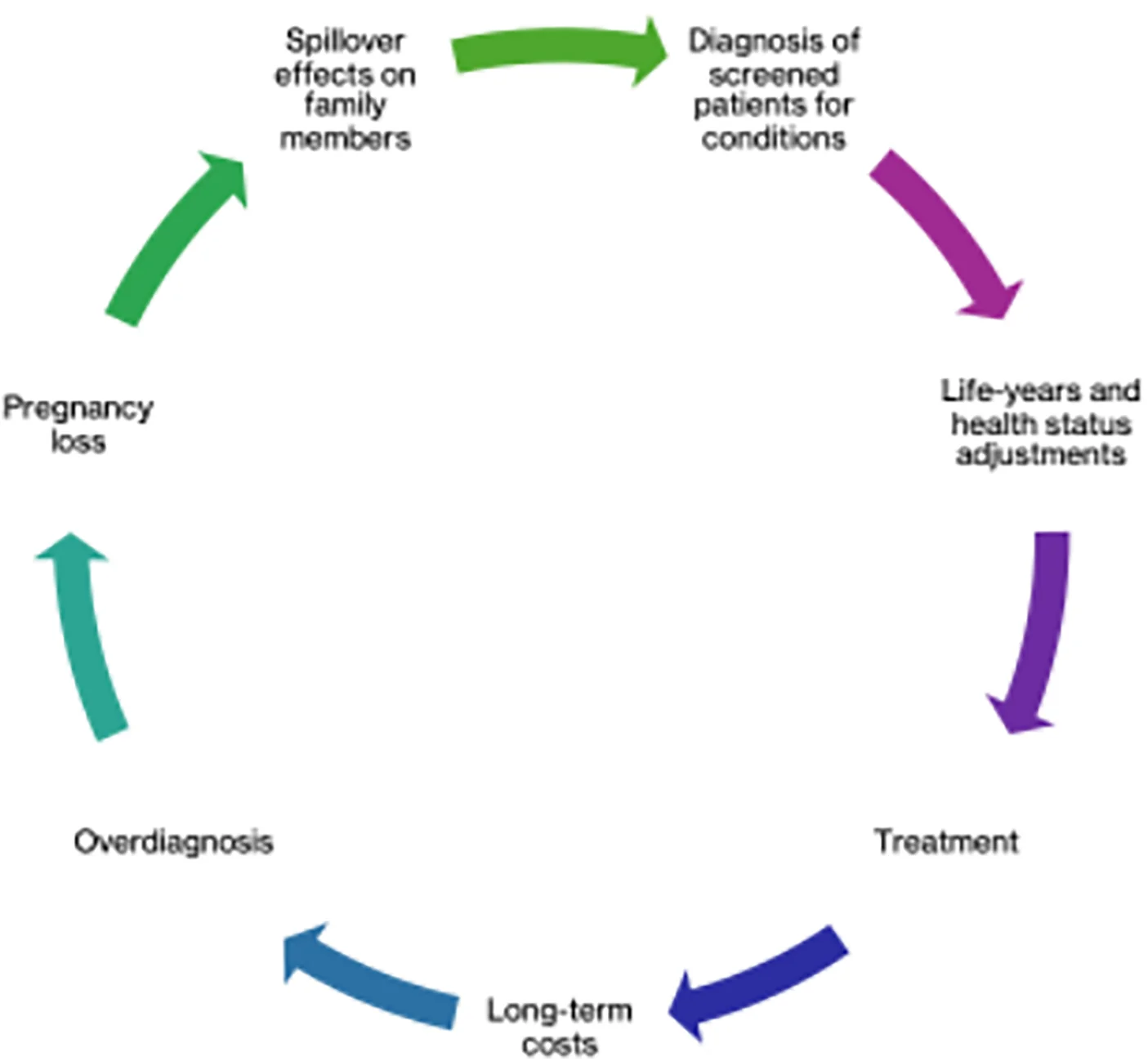
Thematic framework for assessing the harmful effects and benefits of prenatal and neonatal screening programs within health economics evaluations, adapted from Png et al. (20) and Rivero-Arias et al. (19). The graphical layout was redesigned by the authors for the purposes of this review.

Cost effectiveness analysis (ROP-related CEA), cost-utility analysis (treatment/diagnostic techniques and screening), and social burden of disease studies were included, irrespective of publication language. Non-English articles were translated by the first author, a native Italian speaker, and supplemented when necessary by professional translation tools (or using validated online translation tools). The obtained papers were integrated with further relevant articles using the “Similar articles” and “Cited articles” functions of PubMed, as suggested by the John Hopkins University and University College of London websites (12, 13), to expand the number of eligible articles when too few articles were output for one's purposes in the PubMed main menu. The additional publications retrieved through PubMed's “Similar Articles” and “Cited Articles” functions were independently screened by the first author and discussed with the senior author (last author) when eligibility was uncertain. As already described by Topfer et al. and Arber et al., PubMed was used as the main database while Embase was considered as complementary dataset (14, 15) although previous researchers has found that sometimes, multiple references databases beyond PubMed could have a modest impact on maximizing bibliographic retrieval (16). Accordingly, we ran on 6th July 2026 the search strings “economic impact of ROP” and “social cost of ROP” on Embase and Cinahl excluding one potentially relevant EMBASE record because only the conference abstract was available (17). From Embase, papers were excluded because either they were duplicates of PubMed articles or they were not relevant to ROP. From Cinahl, two eligible papers were included excluding the not-relevant papers for the same reasons. Titles and abstracts were initially evaluated for relevance to the review objectives. Full texts were retrieved whenever eligibility was uncertain. Articles were included if they provided original or synthesized evidence on the social burden, economic burden, cost-effectiveness, cost-utility, screening, imaging, telemedicine or treatment of ROP. Publications focused exclusively on clinical efficacy, biological mechanisms or unrelated ophthalmological conditions were excluded. Data extraction was facilitated through Elicit software (18) in order to preliminarily organize bibliographic information and descriptive data extraction. All extracted information was independently verified against the original publications. Accordingly, no AI-generated manuscript text or scientific interpretation was produced. Thereafter, the two groups of papers were analyzed in the Discussion section per topic according to the Rivero-Arias et al.'s thematic framework (Figure 1) (19) because a thematic evaluation constitutes a more feasible approach to manage a large number of papers, including systematic reviews and reports on prenatal and neonatal economics in relation to the benefits and harms of screening programmes in the Organization for Economic Co-operation and Development (OECD) countries (19, 20). Because this work was intentionally designed as a transnational narrative review integrating heterogeneous evidence (cost analyses, policy reports, implementation studies and observational studies), a formal quality appraisal was not performed and we acknowledge it as limitation in the appropriate section.

## Results

On the first screening on PubMed, 5 articles were identified with “ROP neonate social cost” and 23 with “economic impact of ROP”. However, only 3 and 9 articles were included for the first and second search strings, respectively. PDF full texts were downloaded and reviewed thoroughly because they considered ROP cost-utility intervention analysis, economic impact and social burden of disease, as reported in Supplementary Table S1 (*n* = 12) integrated with further three articles retrieved on Embase and Cinahl (21–24) as Supplementary Material. The characteristics of the 15 included studies are summarized in Supplementary Table S1, including study design, country and setting, population, intervention/comparator, economic perspective, time horizon, principal findings, and methodological strength. Particularly, methodological strength was descriptively classified according to the underlying study design (High, Moderate or Low) and was intended to facilitate interpretation of the evidence rather than to represent a formal methodological quality appraisal. Supplementary Table S2 shows the evidence type associated with each paper included in the Supplementary Table S1. The 17 excluded papers were reviews, duplicates of the first search string or clinical and biological research not related to the selection topic criteria mentioned in Methodology. Supplementary Table S3 summarizes the 71 additional publications that were considered relevant to the thematic synthesis after citation tracking through PubMed “Similar Articles” and “Cited Articles” functions. This set of supplementary publications was retained because each article contributed contextual, methodological, policy-related, or intervention-specific evidence relevant to at least one of the predefined thematic domains. Overall, the available evidence was heterogeneous in design and methodological rigor, precluding formal quantitative synthesis.

To enhance its readability, the discussion section will be divided into the following five thematic sections: “screening programs”, “telemedicine and imaging”, “cost effectiveness analysis”, “treatment-related economic burden”, “societal burden and quality of life”.

## Discussion

### Screening programs

Among the publications retrieved through PubMed's “Similar Articles” function from the paper by Zupancic et al., validation of screening guidelines emerged as a major theme together with ROP risk models and postnatal weight gain as predictive approaches (28). Notably, the Western (UK-US; American Academy of Pediatrics, etc.) screening criteria are not suitable for other countries, such as China (25), India (26) and Jordan (27). Moreover, they are not universally known by healthcare professionals across continents (5). Further population cohort research is necessary to revise screening protocols (25) and considering local NICU-specific organizational differences may improve screening (28). To guarantee the meta-ethnographic validity of screening strategy designs, studies should focus on multiple countries and national-level engagement in treatment and screening (29), particularly in resource-limited NICU settings compared with major health centers. Worldwide, neonatologists and pediatric ophthalmologists base their screening activities on national guidelines that may be upgraded annually. Indeed, a notable example is provided by Sweden (30), who used two cohorts from a national quality register (SWEDROP) as a base to assess the applicability of national guidelines and support clinicians in optimizing screening intervals, such as lowering upper age limits by one week.

### Telemedicine and imaging

From a cost-effectiveness perspective, AI-assisted telescreening (31, 32), mobile units (33–35), and wide-field digital imaging (36, 37) as separate strategies have demonstrated favorable economic profiles, even in LMICs. These tools offer scalable, efficient ways to reduce preventable blindness. For example, a Dutch telemedicine service called “Remote Eye Triage”, covering cataracts, diabetic retinopathy (DRP), age-related macular disease (AMD), glaucoma, and dry-eye syndrome (DES), has demonstrated the potential to mitigate such delays (38). Furthermore, studies exploring the cost of inaction—particularly the economic burden of children left blind due to missed or delayed screening—are rare but critically needed. National-level programs such as Saudi Arabia's tele-ROP system (39) and Argentina's policy-driven blindness reduction strategy (40) demonstrate that multisectoral collaboration can deliver effective and sustainable ROP services. Integrating ROP screening into broader neonatal and public health programs—especially via telehealth and AI—can overcome the barrier of specialist shortages. Further, several countries have adopted smartphone-based imaging to enhance ROP diagnosis and documentation (41–44). Collectively, these findings suggest that telemedicine may represent an integral component of sustainable ROP screening programs, but they should be considered cautiously as these studies are often country-centric. Therefore, expanding AI validation studies and training local providers through tele-education (45) are pivotal to advance these approaches in a coordinated manner (healthcare providers, policy makers, industry, etc.). Furthermore, AI implementation will require a pediatric-centric governance centred on transparency, inclusive participation, equitable data practices, and rigorous post-deployment monitoring to ensure safe, responsible integration (56).

### Cost effectiveness analysis

The economic literature consistently suggests that the societal cost of blindness due to ROP—including caregiver burden, lost productivity, and long-term healthcare use—far exceeds the cost of preventive screening and early intervention (46, 47). Overall, the available literature provides encouraging evidence supporting cost-effective screening programmes and preventive interventions. Going forward, health systems could consider adopting country-specific cost-utility models, validating AI tools in diverse populations, and expanding provider training through tele-education initiatives (45). From a policy perspective, ROP may warrant prioritization as a preventable cause of childhood blindness. However, cost-effectiveness and cost-utility data are still lacking, especially for long-term follow-up care, anti-vascular endothelial growth factor therapy vs. laser therapy in different national contexts and the cost of inaction or missed screening.

Despite encouraging findings, important evidence gaps remain, particularly regarding long-term follow-up costs, comparative cost-effectiveness of anti-vascular endothelial growth factor vs. laser therapy across healthcare systems, and the economic consequences of delayed or missed screening. Kana et al. reported that, in their resource-limited setting, intravitreal anti-vascular endothelial growth factor therapy was associated with lower procedural costs than laser therapy because it avoided general anesthesia, prolonged operating room use, and high-care admission. Notwithstanding these results, the authors also acknowledged the need for more frequent follow-up, suggesting that the economic advantage should be interpreted within the local healthcare context (48). Overall, the currently available comparative evidence remains insufficient to support one treatment as economically superior across different healthcare systems. Accordingly, future research should focus on conducting robust economic analyses capable of informing sustainable screening policies across different healthcare systems on robust, country-specific economic evaluations capable of informing sustainable screening policies and equitable access to neonatal eye care across diverse healthcare systems.

### Treatment-related economic burden

Few economic evaluations assess long-term follow-up costs for survivors with sequelae. Similarly, although anti-vascular endothelial growth factor therapies are increasingly used, their cost-effectiveness compared to that of laser treatment varies by setting and remains underevaluated (49, 50). Although evidence remains limited, some studies included in this review suggest that anti- anti-vascular endothelial growth factor therapy may offer economic advantages in selected settings due to reduced procedural requirements and lower anesthesia-related costs. However, the need for prolonged follow-up and concerns regarding late recurrence may offset some of these advantages. Consequently, definitive conclusions regarding the comparative cost-effectiveness of anti-vascular endothelial growth factor vs. laser therapy cannot yet be drawn.

### Societal burden and quality of life

#### Economic burden of prematurity

The following studies do not specifically quantify the economic burden attributable to ROP. Rather, they provide indirect contextual evidence illustrating the broader economic consequences of prematurity, within which ROP represents one important morbidity. The present review underscores the importance of studying the societal cost, cost-effectiveness strategies and screening activities for ROP. However, the international literature is unbalanced, as more low-income countries' dynamics are examined than high-income countries'. AI is often presented as a broadly applicable solution, although its implementation requires careful validation, governance, and context-specific evaluation. Accordingly, evidence supporting its cost-effectiveness remains heterogeneous and context dependent.

Although our paper is not intended to defend Western hospitals to the detriment of non-Western centers, economic cost studies show that NICUs are increasingly expensive and have simultaneous neonatal complications even in high-income countries. In Ontario (Canada) (51) researchers analyzed data on 12,660 infants between 20 and 30 weeks of gestation from the Ontario Healthcare Data Service (2005–2017). Retinopathy of prematurity ranked among the most expensive neonatal morbidities, with an incremental of approximately US$56,000 per infant.

Moreover, in a multinational European cohort study conducted in Belgium, Denmark, Estonia, France, Germany, Italy, the Netherlands, Poland, Portugal, the United Kingdom and Sweden (52), researchers studied economic and societal costs (2016 cost values expressed in euros) at five years of age associated with very preterm birth, including ROP and other neonatal morbidities (IVH, severe respiratory diseases such as BPD, etc.). A lower gestational age was associated with increased mean societal costs of €2755 (*p* < 0.001), €752 (*p* < 0.01) and €657 (*p* < 0.01) for children born at <26, 26–27 and 28–29 weeks, respectively, in comparison to the reference group born at 30–31 weeks. This study illustrates the stratified costs of prematurity-related morbidities across Europe. Supplementary Table S4 summarizes the distribution of direct and indirect costs across participating countries for former preterm infants at 5 years of age (52) considering medical, social and rehabilitation services. Notably, the total social cost varied among European countries.

Even in South Korea, the preterm birth rate and associated medical costs have increased despite the resulting birth decline. In an observational study on hospitalization costs covered by national public insurance for preterm infants (53), a total of 5,312,886 live infants were born, 90,575 of whom were diagnosed with preterm birth hospitalization. The total medical cost per patient almost tripled from $7,390.90 to $20,209.59 from 2008 to 2020; for the extremely preterm group, the total medical cost increased fourfold ($13,961.03 to $55,984.47). These findings support the need for efficient national resource allocation to reduce inequalities in access to neonatal care (51) to avoid the inequalities concerning the economic opportunity for health services as an additional health determinant (54, 55) that could be mitigated by analyzing and implementing economic policies related to health effects.

In a multicenter study of ROP clinical and socioeconomic factors impacting follow-up adherence in Los Angeles Neonatal Intensive Care Units (56), public insurance status and hospital setting were important predictors of incomplete ophthalmologic follow-up after ROP screening, highlighting the influence of socioeconomic factors on continuity of care. In this sense, a global ROP strategy must be context sensitive, economically justified, and built on strong interdisciplinary collaboration to ensure that no infant is blind due to a lack of timely and cost-effective care. Indeed, ROP care requires a team-based approach to identify and address barriers to care, including access to care, health literacy, language barriers, high healthcare utilization, and frequent need for follow-up (57), via a multidimensional family-centered approach. Accordingly, “parents of infants with ROP often face a multitude of social, emotional, and financial factors that can affect adherence to care, and it is important not only for ophthalmologists but also for the entire care team to recognize and align resources to provide the best care possible to prevent vision loss”.

#### Economic burden of blindness and visual impairment

Similarly, the studies discussed below concern the societal costs of blindness and visual impairment irrespective of etiology. They are presented as contextual evidence supporting the potential long-term economic implications of ROP-related blindness rather than direct estimates of ROP-specific costs. Indeed, visual impairment and blindness constitute a noteworthy economic burden for affected individuals, as demonstrated by numerous studies included in recent systematic reviews of the literature (58). These conditions lead to indirect costs, including productivity loss, premature mortality, deadweight loss, early retirement, and family care support—as reported in a study from Germany (59), which showed that the annual societal cost of blindness and visual impairment was €49.6 billion. In that country, these societal costs are significantly greater than those for people with normal vision, highlighting the importance of implementing patient-centered interventions (60).

In the United Kingdom, a prospective cross-sectional study, BCVIS2 (British Childhood Visual Impairment and Blindness Study 2), revealed that the incidence rates of blindness and visual impairment were greater among those in the lowest socioeconomic quintile, as well as in children born preterm or with low birthweight (61). Socioeconomic status was also associated with visual impairment and blindness in a global study involving 190 countries published in *JAMA Ophthalmology* (62).

Across Europe, blindness and moderate-to-severe visual impairment were associated with a substantial societal burden, combined estimated between €25 and €56 billion depending on the economic model adopted (63).

In India, employment loss and caregiver time represented the major contributors to the societal costs of blindness and visual impairment (64).

Unfortunately, blindness and MSVI continue to impose a large global economic burden, as confirmed by another systematic review published in *The Lancet—EClinicalMedicine* (65). The authors propose that reducing and preventing vision loss while implementing strategies to support visually impaired individuals in securing and maintaining employment could generate significant productivity gains. In this context, delays in eye care are associated with increased societal costs and reduced quality of life (QoL) for patients (38).

## Conclusions

This narrative review highlights that ROP should be considered not only a neonatal ophthalmologic condition but also a socioeconomic and public health challenge. The available evidence suggests early screening, context-specific implementation of telemedicine and digital imaging, and multidisciplinary collaboration as key strategies to reduce preventable blindness. However, important evidence gaps remain regarding long-term economic outcomes, treatment-related cost-effectiveness, and country-specific evaluations. Although no universally applicable model currently exists, future research should aim to develop a shared framework for early neonatal eye screening while also addressing social disparities, emerging technological innovations such as AI, health law, and health economics across Europe and North America. Indeed, prematurity-related diseases may be associated with poverty, ethnicity and deprivation (56, 66–70). Healthcare systems may benefit from prioritizing universal and context-specific ROP screening programs, supporting the implementation of validated telemedicine and AI-assisted technologies, investing in workforce training through tele-education, and promoting national registries capable of monitoring clinical and economic outcomes. Such strategies may improve equitable access to care while reducing the long-term societal burden associated with preventable childhood blindness. Future research could address its efforts to conducting robust economic analyses capable of informing sustainable screening policies across different healthcare systems. Viewing ROP through a health economics perspective may help policy-makers prioritize sustainable investments capable of preventing lifelong disability while reducing future societal costs. Ultimately, sustainable investments in ROP prevention may represent not only clinically effective interventions but also economically sound public health strategies.

### Redefining progress in ROP care

Based on the evidence synthesized in this review, we believe that future progress in ROP care should be evaluated not only by reductions in blindness or treatment success, but also by the capacity of healthcare systems to deliver equitable, sustainable, and economically efficient screening and follow-up programmes. Particularly in low- and middle-income countries (LMICs), progress should also be evaluated according to the successful implementation of affordable and feasible screening technologies. While AI and other technological innovations may not always be feasible, particularly in rural or resource-limited settings, economic sustainability and clinical benefits should be balanced. Rather than focusing exclusively on increasingly sophisticated technologies, future efforts should prioritize solutions that can be realistically implemented within local healthcare systems. International collaboration among health economists, policy-makers, engineers and LMIC clinicians may facilitate the development of affordable, sustainable and context-specific strategies without compromising quality of care. Accordingly, future ROP programmes should increasingly be assessed through their economic sustainability, scalability, health-system integration, and long-term societal benefits.

## Limitations

This review has multiple limitations. First, only one bibliographic database (PubMed) was searched, potentially limiting retrieval of relevant studies. Second, additional evidence was identified through citation tracking, which may have introduced selection bias. Third, no formal quality appraisal was performed because of the narrative design. Finally, the included studies were highly heterogeneous in design, healthcare settings, economic perspectives, and outcome measures, precluding quantitative synthesis.
