# Supplementary material for: The social and economic burden of retinopathy of prematurity: screening, treatment, and health policy perspectives in a transnational narrative review
Source: Front Pediatr. 2026 Aug 26;14:1894390. doi: 10.3389/fped.2026.1894390 (PMC13554956; doi:10.3389/fped.2026.1894390)
Supplement: Supplementary file 1 [file Table1.docx]

SUPPLEMENTARY MATERIAL

Tab. 1 - *Eligible papers associated with PubMed’s search strings “ROP neonate social cost” (2012-2025) and “economic impact of ROP” (2003-2025) combined with EMBASE and Cinahl retrieved articles.*

| Authors | Title | Study design | Country | Population | Economic perspective | Comparator | Time horizon | Main results | Methodological strength |
| --- | --- | --- | --- | --- | --- | --- | --- | --- | --- |
| Zupancic et al. | Evaluation of the economic impact of modified screening criteria for retinopathy of prematurity from the Postnatal Growth and ROP (G-ROP) Study | The study design is a microsimulation model using data from the G-ROP study, which is simulation-based. It compares Conventional Screening (CS) and G-ROP criteria, with two phases: "Discharge" for short-term outcomes and "Lifetime" for long-term societal costs and quality of life. | United States | - Population: Preterm infants born in the United States with a gestational age less than 32 weeks. - Cohort size: 78,281 infants. - Gestational age range: 22 to 32 weeks. | - Primary analysis: Third-party payer perspective, focusing on direct medical costs, with a time horizon to first discharge home from hospital. - Lifetime analysis: Societal perspective, considering all costs including family expenses, productivity losses, and educational expenditures, with a lifetime time horizon. | Conventional Screening (CS) and G-ROP Screening | - Primary analysis: Time horizon to first discharge home from hospital - Lifetime analysis: Lifetime time horizon | - G-ROP detected approximately 25 additional infants with Type 1 ROP compared to conventional screening. - This was achieved with 36,233 fewer examinations and in 14,073 fewer infants. - Annual cost savings were approximately $2,931,980 USD in the short run and $12,873,157 USD in the long run. - G-ROP is considered a "dominant" option due to its higher effectiveness and lower costs compared to conventional screening. | *High* |
| Zin et al. | Retinopathy of prematurity screening and treatment cost in Brazil | The study design involved cost analysis, incremental cost analysis, and budget impact analysis using a deterministic decision-tree simulation model based on real-world data from Rio de Janeiro (previous observational study), with sensitivity analyses to test parameter variations. | Brazil | - Total live births in Brazil in 2010: 2,857,554 - Premature infants weighing less than 1,500g: 36,609 - Survived premature infants: 13,355 - Premature infants requiring eye examinations: 13,500 - Premature infants needing laser treatment: approximately 1,000 | - Economic perspective: The study uses cost analysis, incremental cost analysis, and budget impact analysis to evaluate the economic implications of ROP screening and treatment in Brazil. - Unit costs: US$ 18 for each examination, US$ 398 for treatment, and US$ 29 for training. - Estimated cost for all at-risk infants: US$ 80 per infant. - Additional annual costs to SUS: US$ 556 640 for 52% coverage, US$ 856 320 for 80% coverage, and US$ 1.07 million for 100% coverage. - Incremental cost to SUS: 0.61% to 0.98% of NICU hospitalization costs. | The comparator in this study is the cost of hospitalization without ROP screening and treatment, using two different estimates of hospitalization daily costs (US$ 240 and US$ 389) as a basis for comparison. | one year | - Estimated cost of ROP diagnosis and treatment for all at-risk infants: US$ 80 per infant. - Additional annual costs to the SUS: US$ 556 640 for 52% coverage, US$ 856 320 for 80% coverage, US$ 1.07 million for 100% coverage. - Cost per examination: US$ 18, with staff costs being the main driver. - Cost per treatment: US$ 398, with equipment costs being significant. - Incremental cost to the SUS: less than 1% of NICU hospitalization costs. | High |
| Neves et al. | Cost-Utility Analysis of Wide-Field Imaging as an Auxiliary Technology for Retinopathy of Prematurity Care in Brazil | Cost-utility analysis using a deterministic decision-tree simulation model with one-way sensitivity analysis and Monte Carlo probabilistic simulation. | Brazil | Preterm infants with GA <32 weeks or BW ≤1,500 g born in governmental maternities in Brazil | The economic perspective in this study is from the Brazilian Unified Health System's (SUS) viewpoint, focusing on cost-utility analysis using a deterministic decision-tree simulation model to estimate incremental cost per quality-adjusted life-year (QALY) gained. The study calculates the ICUR as US $1,746.99/QALY for the combined screening strategy and discusses the willingness-to-pay threshold, although Brazil lacks an established threshold. | Indirect binocular ophthalmoscopy (IBO) is the comparator in this study, as it is the standard practice against which the combination strategy of wide-field imaging (WFI) with IBO is compared. | Lifetime | - The combined strategy of wide-field imaging with indirect binocular ophthalmoscopy is cost-effective for ROP screening. - It enhances screening coverage, particularly in areas lacking ROP experts. - Visual impairment rates are reduced by 36% compared to traditional ophthalmoscopy. - Approximately 455 visually impaired children could be avoided annually in Brazil. - The ICUR for the combined strategy is US $1,746.99/QALY. - The combined strategy is 100% likely to be cost-effective at a willingness-to-pay threshold of US $1,800/QALY. | *High (or moderate?)* |
| Dave et al. | The Societal Burden of Blindness Secondary to Retinopathy of Prematurity in Lima, Peru | Cost-of-illness study with retrospective observational data collection for direct costs and economic analysis for indirect costs | Peru | Neonates and children affected by retinopathy of prematurity (ROP) in Lima, Peru; estimated 4174 children currently affected by ROP. | - Direct cost of laser treatment: $2496 per child - Indirect cost saving: $197 753 per child - Societal lifetime cost saving per child: $195 257 - Generational cost savings: approximately $516 million - Cost-effectiveness: $69/quality-adjusted life year | Natural history of ROP (no treatment) vs evidence-based treatment (laser treatment) | 20 years | - Direct cost of laser treatment per child: $2496 - Indirect cost saving per child: $197 753 - Societal lifetime cost saving per child: $195 257 - Equivalent employment: 24 educated Peruvians per year - Generational cost savings: $516 million - Cost-effectiveness: $69/quality-adjusted life year - National lifetime burden: $516 766 244 | High |
| Lai and Lorch | Healthcare Costs of Major Morbidities Associated with Prematurity in US Children's Hospitals | Retrospective cohort analysis - Population: Infants born at 24-30 weeks of gestation - Data source: Pediatric Health Information System (PHIS) - Inclusion criteria: Admitted to children's hospitals within the first three postnatal days - Data collection: Billing charges used to estimate healthcare costs |  | Infants born at 24-30 weeks of gestation, admitted to children's hospitals in the US, without major congenital anomalies, and with specific morbidities associated with prematurity (BPD, IVH, NEC, ROP, and NI). | - The study provides a comprehensive cost evaluation of major morbidities associated with prematurity. - The use of PHIS for cost estimation and inflation to 2018 US$ indicates a focus on economic data. - Quantile regressions were used to examine incremental costs, which is valuable for economic evaluations. - The paper highlights the economic impact of prematurity, with severe BPD, surgical NEC, and severe ROP being the costliest morbidities. - The study provides robust cost estimates for economic evaluations of neonatal interventions. | The comparator in the study is the absence or lower severity of the major morbidities associated with prematurity (BPD, IVH, NEC, ROP, and NI). | The time horizon for this study is the initial hospitalization period, specifically from birth to discharge home after 34 weeks PMA or survival to 36 weeks PMA, with data collected between 2009 and 2018. | - Surgical NEC, severe ROP, and severe BPD are the costliest morbidities. - Median total costs: Surgical NEC ($430,860), severe ROP ($413,825), severe BPD ($399,495). - Adjusted median incremental costs: Surgical NEC ($48,621), severe BPD ($35,773), severe ROP ($22,561). - These morbidities have increasing incremental costs at higher total cost percentiles. | High |
| Kelkar et al. (a) | A mobile team for screening of retinopathy of prematurity in India: Cost -effectiveness, outcomes, and impact assessment | Prospective observational study. Public health intervention study. | India | 102 preterm/underweight infants aged <34 weeks of gestation and/or birth weight ≤1700 g, admitted in five NICUs in Pune, India between 2013 and 2015. | The economic perspective in the paper focuses on the cost-effectiveness of a mobile screening model for ROP in India, detailing unit costs for screening (US $198.9), identifying (US $596.7), and managing (US $4137.4) ROP cases. It emphasizes affordability compared to other methods like telemedicine and considers the societal burden of blindness. | Telemedicine | 3 years (2013-2015) | - Prevalence of ROP: 32% (95% CI: 23.2-41.5) - ROP stage I prevalence: 75% - Infants helped by the model: 4 with advanced stages of ROP - Unit costs:  - ROP screening: US $ 198.9  - Identifying one child with ROP: US $ 596.7  - Addressing visual disability due to ROP: US $ 4137.4 | High (the study is well-designed with ethical approval, a significant sample size, adherence to international guidelines, and a focus on cost-effectiveness and sustainability) |
| Gilbert and Todd | Impact of COVID-19 pandemic on retinopathy of prematurity services in low resource settings | Mixed-methods observational study with a cross-sectional design, It involved online survey (eSurvey) with closed and open-ended questions, followed by interviews with a subset of participants for qualitative insights. | Multinational cohort | - Geographic Location: Low- and middle-income countries - Number of Countries: 32 - Economic Classification: 56% UMICs, 42% LMICs, 2% LICs - Professional Role: Ophthalmologists providing ROP services - Number of Participants: 184 survey participants, 15 interviewees | The economic perspective in the paper highlights the impact of the COVID-19 pandemic on households and the recession, which affects access to ROP services. It emphasizes the economic burden on parents, particularly in LMICs and LICs, and suggests that health insurance coverage could alleviate this burden. The economic constraints also affect healthcare providers' ability to access necessary treatment modalities. | Pre-pandemic state of ROP services | January 15 to March 12, 2021 (survey), August 2021 (interviews) | - A moderate to severe impact was reported by 13% for screening, 9% for treatment, and 16% for vitreoretinal surgery. - 22% thought services would take a year to recover. - Many reported fewer neonatal admissions and more ROP blind infants once restrictions lifted. - The pandemic had a variable impact on ROP services, with some countries experiencing a significant impact. - Half of the participants continued to provide ROP services despite challenges. | *Moderate (the study uses a mixed-methods approach and includes both surveys and interviews, but has limitations such as low response rates)* |
| Kelkar et al. (b) | Mobile unit for retinopathy of prematurity screening and management at urban Neonatal Intensive Care Units: Outcomes and impact assessment | Prospective observational study with a cohort-like design | India | Preterm infants aged <33 weeks and/or birth weight 1250 g or less, admitted in five NICUs in Pune, India; total of 104 infants | - Total cost for 3-year project: US $32,285 - Unit cost per child: US $310 - Unit cost to identify one child with ROP: US $950 - Unit cost to address visual disability due to ROP: US $6,500 - Mobile screening is considered feasible and cost-effective in urban areas with limited resources. | Not mentioned (the paper does not specify a comparator or control group for the mobile unit) | 3 years | - Incidence of ROP: 32.7% (95% CI: 23.7-41.7) - Grades of ROP: Detailed in Table 1 - Intervention outcomes: 29 monitored without intervention, 4 treated with laser, 1 with pars plana vitrectomy - Prevention of blindness: Mobile unit helped prevent blindness in 5 eyes with advanced ROP - Cost-effectiveness: Total cost over 3 years was Indian Rupees 1,872,486 (US $32,285); unit cost per screening was US $310; unit cost to identify one child with ROP was US $950; unit cost to address visual disability was US $6500 - Feasibility and cost-effectiveness: Mobile screening is a feasible and cost-effective strategy for ROP detection and intervention in urban areas with limited resources | Moderate |
| Rothschild et al. | The Economic Model of Retinopathy of Prematurity (EcROP) Screening and Treatment: Mexico and the United States | The study design is a prospective, cost-effectiveness, cost-utility, and cost-benefit analysis using a decision analytic model with sensitivity analysis. It compares current screening and treatment practices with an ideal national program. | Mexico and United States | The population in this study refers to premature infants at risk for retinopathy of prematurity (ROP) in Mexico and the United States, particularly those weighing less than 1500g or born at less than 30 weeks' gestational age. The study extrapolates data to estimate national impact, but a specific numerical population size is not provided. | The economic perspective in this study involves a comprehensive analysis of the cost-effectiveness, cost-utility, and cost-benefit of retinopathy of prematurity (ROP) screening and treatment. It assesses both direct and indirect costs from a societal perspective, providing specific economic figures for Mexico and the United States. The model is designed to be applicable to any country or region, with a focus on cost savings and societal benefits. | Current levels of screening and treatment of ROP | The intervention timeframe is the first year of life for a premature infant, and the analytic horizon is the lifetime of the affected individual. | - An ideal national ROP screening and treatment program is highly cost-saving in both Mexico and the United States. - The incremental net benefit of an ideal ROP program over current practice is $5556 per child ($206 574 333 annually) in Mexico and $3628 per child ($205 906 959 annually) in the United States. - Universal screening is cost-saving in both countries, with a negative ICER value. - There is a high probability (91% in Mexico, 82% in the United States) that a national screening program would be cost-effective or cost-saving. | High |
| Kana et al. | The efficacy of intravitreal antivascular endothelial growth factor as primary treatment of retinopathy of prematurity: Experience from a tertiary hospital | Retrospective observational study | South Africa | - Location: St John Eye Hospital, Johannesburg, South Africa - Time period: 2013 - 2015 - Total screened: 1,911 - Treated: 23 (1.2%) - Mean birth weight: 1,074 g - Mean gestational age: 28 weeks - Gender: 13 males, 10 females | - Cost of IVB injection: ZAR12 529.83 - Cost of laser therapy: ZAR25 916.03 - Difference mainly due to theatre time and high-care monitoring for laser therapy - IVB requires more follow-up visits but is overall less expensive | Laser therapy | 3 years (2013-2015) for data collection; follow-up period up to 60 weeks' gestational age or mean follow-up of 9 months (range 1-18 months) | - 95.3% of eyes showed complete regression or non-progression of ROP. - Two patients experienced progression leading to retinal detachment. - IVB is more cost-effective than laser therapy, with a cost difference of approximately ZAR13,386.20. - IVB is effective and safe for the treatment of ROP. | Low |
| Moitry et al. ´ | Effectiveness and efficiency of tele-expertise for improving access to retinopathy screening among 351 neonates in a secondary care center: An observational, controlled beforeafter study | Observational, controlled before-after study |  | - Location: Centre Hospitalier Sud Francilien (CHSF), a secondary care center in the Paris Region - Criteria: Infants born before 33 weeks of gestational age and/or with a birth weight less than 1500 grams, without cerebral malformations - Total recruited: 351 infants at CHSF - Control group: Port-Royal, a university tertiary care center with 217 and 269 infants in the before and after periods | The economic perspective in the study involved assessing the costs associated with implementing a tele-expertise program for ROP screening. The average cost per examination increased from €337 to €353, with overall costs being almost the same for both standard and tele-expertise procedures. The study suggests potential future cost reductions through equipment price negotiations or training. | Port-Royal, a university tertiary care center with on-site ophthalmologists | - Data collection periods: January 1, 2012, to December 31, 2012, and January 1, 2014, to March 31, 2015 - Economic analysis time horizon: 5 years | - Implementation of tele-expertise increased the proportion of examinations conducted in accordance with guidelines by 57.3% (from 3.8% to 61.1%). - Compared to the control group, tele-expertise improved the proportion of appropriately screened infants (57.5% vs. 43.1%). - The average cost per examination increased slightly from €337 to €353. - Infants undergoing tele-expertise experienced higher pain levels during examinations (median score 5.5/10 vs. 2.0/10). | Moderate |
| Naguib et al. | Regionally specific economic impact of screening and treating  retinopathy of prematurity in middle-income societies in the  Philippines | Economic modeling study using cost-effectiveness, cost-benefit, and cost-utility analysis with a decision tree analytic model and sensitivity analysis. | The Philippines | Not mentioned (the abstract does not provide specific information about the population size or demographic characteristics) | The economic perspective in this study involves using the Economic Model for Retinopathy of Prematurity (EcROP) to conduct cost-effectiveness, cost-benefit, and cost-utility analyses. The study focuses on estimating economic effects, incremental cost-effectiveness ratios, and monetary benefits to evaluate the cost-effectiveness of a national ROP screening and treatment program compared to current policies. The findings indicate substantial societal cost savings and a significant national annual net benefit, highlighting the economic benefits of implementing such a program. | Current policy | 1 year | - The incremental cost-effectiveness ratio for a national ROP program is strongly favorable and represents substantial societal cost savings. - The per-child annual monetary benefit is $2,627. - The national annual net benefit is $64,320,692. - The program is cost-saving and cost-effective, reducing childhood blindness. | High |
| Smith et al. 2026 (EMBASE) | Oxygen blenders for prevention of retinopathy of prematurity are highly cost-effective in Uganda | Cost-utility analysis (model-based) | Uganda | Preterm infants at risk of ROP. | Health system / cost-utility (DALYs) | Standard care (unblended oxygen) | Lifetime (58 years) | Implementation of oxygen management equipment was highly cost-effective, reducing severe visual impairment and DALYs at low cost. | High |
| Alizadeh et al., 2026 | Cost–utility analysis of retinopathy of prematurity screening strategies of universal referral, RetCam telemedicine, and direct examination in Iran: a markov model approach | Prospective cost-utility analysis (Markov model) | Iran | Preterm infants eligible for ROP screening | Societal | Direct ophthalmologic examination | Lifetime | RetCam telemedicine was the most cost-effective screening strategy. | High |
| Chatmethakul et al., 2026 | Impact of Telemedicine on Retinopathy of Prematurity Screening at a Level II Neonatal Intensive Care Unit in Southwest Oklahoma: A 6-Year Experience | Retrospective observational study | USA | Lower-risk premature infants undergoing Tele-ROP screening | Hospital/family cost perspective | None reported. | 6 years (2017–2023) | Telemedicine expanded access, reduced travel costs, and safely delivered ROP screening | Moderate |
| Baek et al. 2026 | Cumulative Burden of Neonatal Morbidities and Its Impact on Medical Costs and Length of Stay in Preterm Infants: A Nationwide Study in Korea | Retrospective nationwide cohort | Korea | 41,338 preterm infants | Healthcare system | Increasing burden of neonatal morbidities | 2020–2023 hospitalization | Increasing neonatal morbidities were associated with progressively higher medical costs and longer NICU stay. | High |
| Mladenovich et al., | Retrospective observational study |  | Serbia | Children with visual impairment born before and during the war period | Societal | Pre-war vs war cohorts | Birth cohorts before 1990 vs 1990–2000 | Economic sanctions and war were associated with a higher burden of ROP-related blindness. | Moderate |

Tab. 2 – Eligible studies from PubMed, Embase and Cinahl per evidence type.

| **Study** | **Evidence Type** |
| --- | --- |
| Zupancic et al. | **Direct ROP evidence** |
| Zin et al. | **Direct ROP evidence** |
| Neves et al. | **Direct ROP evidence; indirect contextual evidence (prematurity)** |
| Dave et al. | **Indirect contextual evidence (visual impairment)** |
| Lai & Lorch | **Direct ROP evidence** |
| Kelkar et al. (mobile team) | **Direct ROP evidence; indirect contextual evidence (visual impairment)** |
| Moitry et al. (COVID-19) | **Direct ROP evidence; indirect contextual evidence (visual impairment)** |
| Kelkar et al. (mobile NICU) | **Direct ROP evidence** |
| Rothschild et al. (EcROP) | **Direct ROP evidence; indirect contextual evidence (visual impairment)** |
| Kana et al. | **Direct ROP evidence** |
| Naguib et al. (tele-expertise) | **Direct ROP evidence** |
| Gilbert et al. (Philippines) | **Direct ROP evidence** |
| Smith et al. (Uganda, 2026) | **Direct ROP evidence; indirect contextual evidence (visual impairment)** |
| Chatmethakul et al. | **Direct ROP evidence** |
| Alizadeh et al. | **Direct ROP evidence; indirect contextual evidence (visual impairment)** |
| Mladenovich et al.  Baek et al. | **Direct ROP evidence**  **Direct ROP evidence** |

*Tab. 3 – Further 71 papers found with similar articles and cited articles on PubMed’s search string “ROP neonate social cost” papers (2012-2025) and “economic cost of ROP” (2003-2025).*

| Author(s) | Country | Topic |
| --- | --- | --- |
| Morrison SL et al. | United States of America | AI-based Screening |
| Young BK et al. | India | Smartphone Screening + AI |
| Mohammadi SF et al. | Iran | Tele-screening |
| Haefeli LM et al. | Brazil | Portable Imaging |
| de Sant’Ana VR et al. | Brazil | Smartphone Imaging |
| Ortiz A et al. | Mexico and Argentina | AI-Enabled Screening |
| Al-Khaled T et al. | Argentina, Armenia, Brazil  China, Colombia, Costa Rica, Cuba, Egypt, India,  Indonesia, Iran, Mexico, Mongolia. Nicaragua, Nigeria, Pakistan, Peru, South Africa, Sudan, Vietnam (systematic review) | Telemedicine & Technology |
| Barrero-Castillero A et al. | United States of America (review) | Telehealth & Workforce |
| Alder CJ et al. | Rwanda | National Screening |
| Del Busto Wilhelm E et al. | Guatemala | Screening vs. Full Management |
| Raj S, Kumar P (2021a) | India (Northern region) | Educational Materials |
| Patel SN et al. | Mexico | Tele-education |
| Pirelli A et al. | Italy (Clinical Guideline Review) | Pain Management |
| Asturias AL et al. | Guatemala | Telemedicine Screening |
| Kim E et al. | Australia (Queensland) | Telemedicine Models |
| Zhang RH et al. | Worldwide (including Asia and Africa) | Burden of Disease |
| Isaac M et al. | Canada | Telemedicine Screening |
| Vinekar A et al. (2017) | India | Mobile Screening Unit |
| Hariharan L et al. | United States of America | Public Health Policy |
| Kamholz KL et al. | United States of America | Early Treatment |
| van den Akker-van Marle ME et al. | The Netherlands | Risk-Based Screening |
| Al Amro SA et al. (2022a) | Saudi Arabia | National Screening Program |
| Pillay T et al. | United Kingdom (England) | Neonatal Service Optimization |
| Chen J et al. | Multinational (Systematic Review & Meta-analysis) | Anti-VEGF vs. Laser |
| Ortiz-Seller A et al. | Spain (international network meta-analysis) | Treatment Comparison |
| Linghu D et al. | China | Anti-VEGF vs Laser |
| Albalawi HB et al. | Saudi Arabia (Tabuk City) | Late-stage Presentation |
| Moin M et al. | Pakistan | Service Burden |
| Nassar MM | Egypt | Screening Protocol Gap |
| Padhi TR et al. (2024) | India (Eastern region) | Videoconferencing ROP |
| Yu TY et al. | Australia (Queensland) | Remote Area Screening |
| Vinekar A et al. (2015) | India | Telemedicine Outreach (KIDROP) |
| Wintergerst MWM et al. | Germany | Smartphone vs Traditional Imaging |
| Bhatia SK et al. | United States of America (review) | US ROP Care Costs |
| Castillo-Riquelme MC et al. | United Kingdom | Digital Screening |
| Smith AF et al. | United States of America, United Kingdom and Canada(Systematic review) | Economic Evaluations |
| Lee SK et al. | Canada | Screening Guidelines |
| Fleck BW et al. | United Kingdom | UK Screening Guidelines |
| Vinekar A et al. (2012) | India | Enrollment Program (REDROP) |
| Alda E et al. | Argentina | National ROP Program |
| Goyal A et al. (2019a) | India | ROP Screening & Treatment |
| Choudhary RA et al. | Pakistan | Smartphone Fundus Imaging |
| Mehta P et al. | India | Outborn Screening Rates |
| Binenbaum G et al. | United States of America | CHOP ROP Model |
| Weaver DT et al. | United States of America | Remote NICU Screening |
| Joish VN et al. | United States of America | Preschool Vision Screening |
| Fijalkowski N et al. | United States of America | SUNDROP Telemedicine |
| Padhi TR et al. (2020) | India (Eastern Region) | District-Level ROP Model |
| Maka E et al. | Hungary | Telemedicine Screening |
| Desurmont MG et al. | France | Digital Imaging Implementation |
| Kleberg et al. | Sweden | Pain Management in Screening |
| Puri S et al. | India (Policy Commentary) | Neglected Screening Issue |
| Ali E et al. | Canada | WINROP Algorithm |
| Zepeda-Romero LC, Gilbert C | Mexico | NICU Resource Limits |
| Nkanga ED et al. | Nigeria | Screening Launch in Nigeria |
| Tram JS et al. | Australia | Workload After Digital Imaging |
| Ademola-Popoola DS et al. | Nigeria | Severe ROP Blindness |
| Dabir S et al. | India (Review) | Incidental Findings |
| Chow LC et al. | United States of America | Oxygen Practice Impact |
| Al Amro SA et al. (2022b) | Saudi Arabia | National Telemedicine Program |
| Chang KM et al. | Malaysia | ROP in Blind Schools |
| Raj S et al. (2021b) | Educational Tool Development | Training Materials |
| Yang Q et al. | China | China Screening Criteria |
| Yang B et al. | China | Laser Outcomes |
| Quinn GE et al. | United States of America and Canada | ROP Incidence and Early Course |
| Goyal A et al. (2019b) | Technology Pilot | Smartphone Wide-field Imaging |
| Gilbert C et al. | India | Public Health Integration |
| Norman M et al. | Sweden | Visual Disability Outcomes |
| Fielder AR et al. | United Kingdom | UK Screening Guidelines |
| Bain LC et al. | United States of America | ROP Screening Challenges |
| Arnesen L et al. | Argentina, Brazil, Colombia, Costa Rica, Cuba, Dominican Republic, El Salvador, Mexico, Nicaragua, and Panama. | Latin America/Caribbean Prevalence |

*Tab. 4* ***-*** *Mean costs during the fifth year of life by cost category and by country; mean (SE) (€, 2016 prices), by Kim* et al*., 2022. We used ISO codes for indicating the pertaining country. BEL: Belgium; DNK: Denmark; EST = Estonia; FRA = France; DEU = Germany; ITA = Italy; NLD = The Netherlands; POL = Poland; PRT = Portugal; GBR = Great Britain; SWE = Sweden.*

|  |  |  | **BEL** | **DNK** | **EST** | **FRA** | **DEU** | **ITA** | **NLD** | **POL** | **PRT** | **GBR** | **SWE** |
| --- | --- | --- | --- | --- | --- | --- | --- | --- | --- | --- | --- | --- | --- |
| Direct medical costs | Hospital outpatient services | Pediatrician | 17.29 (2.71) | 11.57 (2.80) | 18.39 (3.88) | 13.88 (0.93) | 23.04 (2.05) | 52.84 (4.52) | 5.24 (1.27) | 273.07 (30.34) | 34.13 (3.07) | 58.48 (9.95) | 106.10 (24.26) |
|  |  | A&E | 1.80 (0.30) | 8.78 (2.08) | 9.19 (2.04) | 37.24 (4.46) | 32.74 (5.17) | 17.26 (1.66) | 16.05 (3.36) | 5.87 (1.01) | 46.90 (3.49) | 32.68 (5.75) | 72.05 (15.11) |
|  |  | Neurologist | 4.81 (1.05) | 1.42 (0.56) | 26.11 (18.45) | 5.58 (3.13) | 2.50 (0.84) | 4.09 (0.54) | 1.57 (0.91) | 30.76 (5.99) | 3.90 (0.82) | 0.22 (0.05) | 32.61 (8.99) |
|  |  | Ear, nose and throat (ENT) | 5.55 (0.73) | 21.55 (3.25) | 8.94 (1.83) | 8.97 (1.36) | 7.75 (0.93) | 7.59 (1.09) | 8.47 (1.78) | 18.71 (3.74) | 18.81 (1.79) | 5.21 (0.70) | 33.15 (7.62) |
|  |  | Ophthalmologist | 6.78 (2.01) | 9.62 (1.97) | 15.26 (2.42) | 23.53 (2.64) | 24.32 (2.16) | 14.43 (1.92) | 18.50 (7.20) | 29.54 (4.47) | 19.85 (2.37) | 5.29 (0.51) | 65.68 (15.51) |
|  |  | Speech therapist | 111.23 (25.45) | 16.56 (5.77) | 190.55 (43.48) | 200.79 (22.06) | 89.49 (12.58) | 46.88 (5.41) | 52.07 (14.51) | 596.64 (102.30) | 52.44 (7.81) | 29.50 (8.62) | 164.18 (68.02) |
|  |  | Psychologist | 9.71 (3.45) | 7.66 (3.31) | 92.57 (41.37) | 22.75 (4.24) | 10.47 (8.19) | 7.45 (2.08) | 1.62 (1.10) | 244.65 (72.02) | 16.66 (3.94) | 2.59 (1.45) | 39.20 (14.20) |
|  |  | Psychiatrist | 0.63 (0.25) | 0.00 (0.00) | 0.55 (0.41) | 17.83 (7.79) | 0.72 (0.51) | 2.67 (0.68) | 0.85 (0.67) | 2.78 (1.66) | 1.50 (0.41) | 12.19 (9.35) | 39.84 (21.03) |
|  |  | Physiotherapist | 76.46 (13.57) | 52.28 (23.09) | 71.28 (22.42) | 105.92 (13.90) | 25.73 (6.51) | 72.00 (8.59) | 37.95 (12.63) | 451.52 (102.28) | 173.12 (43.52) | 51.95 (16.22) | 47.73 (20.70) |
|  |  | Respiratory specialist | 2.30 (0.65) | 1.96 (0.85) | 6.62 (1.45) | 4.88 (0.74) | 0.75 (0.22) | 3.07 (0.44) | 2.82 (1.10) | 120.04 (19.84) | 22.60 (13.85) | 4.37 (2.21) | 8.58 (1.83) |
|  |  | Total hospital outpatient services | 236.57 (35.46) | 131.38 (28.21) | 439.46 (80.94) | 441.37 (35.27) | 217.51 (22.04) | 228.28 (16.72) | 145.12 (28.12) | 1773.58 (253.39) | 389.91 (51.48) | 202.49 (38.89) | 609.11 (118.88) |
|  | Hospital inpatient services |  | 71.54 (17.82) | 49.54 (37.10) | 29.37 (10.08) | 120.55 (21.01) | 254.79 (54.47) | 683.28 (127.62) | 58.15 (18.95) | 78.75 (17.88) | 66.79 (17.65) | 34.14 (9.33) | 202.85 (72.40) |
|  | Community health and social services | GP | 24.10 (2.13) | 31.74 (3.69) | 18.44 (2.16) | 39.73 (2.97) | 7.99 (1.51) | 3.48 (0.61) | 16.55 (1.97) | 148.38 (20.04) | 12.66 (1.30) | 25.33 (2.25) | 34.37 (4.70) |
|  |  | Dietician | 0.44 (0.25) | 1.71 (0.75) | 0.55 (0.41) | 0.19 (0.19) | 0.39 (0.17) | 0.77 (0.29) | 0.93 (0.47) | 1.32 (0.70) | 1.09 (0.31) | 3.60 (0.83) | 11.11 (3.32) |
|  |  | Health visitor | 0.16 (0.10) | 2.28 (0.85) | 14.51 (4.44) | 0.00 (0.00) | 0.78 (0.39) | 7.24 (5.39) | 1.49 (1.11) | 2.81 (1.28) | 6.50 (3.45) | 2.26 (0.72) | 0.00 (0.00) |
|  |  | School nurse | 4.86 (1.84) | 0.00 (0.00) | 0.67 (0.26) | 0.00 (0.00) | 0.09 (0.06) | 0.01 (0.01) | 3.60 (0.65) | 1.98 (0.57) | 1.54 (0.43) | 6.96 (1.59) | 0.00 (0.00) |
|  |  | Physiotherapist | 37.71 (13.79) | 13.20 (5.75) | 10.59 (4.56) | 29.19 (20.19) | 99.22 (15.28) | 1.46 (0.75) | 2.87 (1.77) | 309.51 (81.45) | 34.22 (6.45) | 47.99 (20.68) | 135.62 (99.96) |
|  |  | Early childhood intervention | 15.28 (9.37) | 23.92 (20.13) | 11.71 (10.95) | 0.00 (0.00) | 21.15 (5.06) | 16.88 (5.04) | 0.52 (0.52) | 243.59 (91.02) | 100.25 (17.59) | 886.91 (416.92) | 60.28 (19.01) |
|  |  | Others | 1.83 (0.32) | 2.32 (0.66) | 12.99 (1.75) | 0.47 (0.14) | 3.69 (0.48) | 0.00 (0.00) | 9.10 (0.98) | 46.88 (4.39) | 6.61 (0.79) | 1.52 (0.30) | 108.17 (14.88) |
|  |  | Total community health and social services | 84.38 (17.66) | 75.16 (21.34) | 69.45 (13.18) | 69.58 (20.40) | 133.32 (17.42) | 29.84 (7.62) | 35.06 (3.45) | 754.47 (155.87) | 162.86 (23.67) | 974.57 (424.11) | 349.55 (106.27) |
| Direct nonmedical costs | Equipment costs^a^ |  | 16.48 (7.77) | 142.07 (48.09) | 29.10 (12.18) | 14.79 (2.82) | 7.57 (2.40) | 23.82 (10.17) | 6.90 (2.96) | 207.28 (73.51) | 18.87 (4.43) | 12.40 (7.70) | 202.25 (180.00) |
|  | Out-of-pocket family expenses |  | 23.52 (8.09) | 61.97 (19.23) | 172.80 (103.13) | 20.09 (6.36) | 15.55 (4.80) | 162.52 (37.49) | 9.75 (5.64) | 729.97 (164.84) | 84.49 (19.02) | 28.48 (17.81) | 1419.72 (851.16) |
| Indirect costs | Valuation of time off work |  | 587.10 (165.41) | 729.22 (314.42) | 732.75 (148.67) | 220.53 (48.17) | 223.18 (80.46) | 617.19 (111.02) | 252.87 (174.50) | 835.54 (239.52) | 734.35 (149.80) | 302.42 (91.20) | 954.93 (423.13) |
| Total societal costs^b^ |  |  | 1019.59 (192.66) | 1189.33 (337.22) | 1472.92 (219.72) | 886.91 (78.14) | 851.92 (113.95) | 1744.93 (201.10) | 507.85 (197.22) | 4379.59 (604.41) | 1457.28 (205.51) | 1554.51 (500.28) | 3738.41 (1122.62) |

*GP* general practitioner, *A&E* accident and emergency.

^a^These costs include any special equipment related to infants, such as wheelchairs, braces, hearing aids, or shoes.

LIST OF SIMILAR/CITED ARTICLES IN TAB. 2).

*NEVES*

*SIMILAR*

Morrison SL, Dukhovny D, Chan RVP, Chiang MF, Campbell JP. Cost-effectiveness of Artificial Intelligence-Based Retinopathy of Prematurity Screening. JAMA Ophthalmol. 2022 Apr 1;140(4):401-409. doi: 10.1001/jamaophthalmol.2022.0223. PMID: 35297945; PMCID: PMC8931675.

Young BK, Cole ED, Shah PK, Ostmo S, Subramaniam P, Venkatapathy N, Tsai ASH, Coyner AS, Gupta A, Singh P, Chiang MF, Kalpathy-Cramer J, Chan RVP, Campbell JP. Efficacy of Smartphone-Based Telescreening for Retinopathy of Prematurity With and Without Artificial Intelligence in India. JAMA Ophthalmol. 2023 Jun 1;141(6):582-588. doi: 10.1001/jamaophthalmol.2023.1466. PMID: 37166816; PMCID: PMC10176185. (SIMILAR ARTICLE DA MORRISON) METODO COSTO-EFFICACE.

Mohammadi SF, Rahban A, Darabeigi S, Salimi N, Farahani A, Lashay A, Alinia C. Cost-effectiveness analysis of tele-retinopathy of prematurity screening in Iran. Int J Ophthalmol. 2021 Apr 18;14(4):560-566. doi: 10.18240/ijo.2021.04.13. PMID: 33875948; PMCID: PMC8025162.

(ha citato Neves; ha citato Zin) Haefeli LM, Neves LM, Zin A, Costa ACC, Farias Meira de Vasconcelos Z, Pinto M. Portable wide-field digital imaging for screening of neonatal visual impairment causes in Rio de Janeiro, Brazil: a budget impact analysis. BMJ Open. 2022 Jun 10;12(6):e056498. doi: 10.1136/bmjopen-2021-056498. PMID: 35688584; PMCID: PMC9189816. (GIUSTIFICARE L’INTRODUZIONE DELL’IMAGING DIGITALE CON DISPOSITIVI PORTATILI ANCHE TRAMITE (citato da Neves) de Sant'Ana VR, do Vale Lúcio KC, Fogaça AJ, Bonini NB, Jorge EC. Smartphone-based retinal camera modified for ROP screening. Int J Retina Vitreous. 2025 Oct 14;11(1):108. doi: 10.1186/s40942-025-00725-x. PMID: 41088492; PMCID: PMC12522991 e GIUSTIFICARE AI Ortiz A, Patiño S, Torres J, Mármol J, Serafin C, Dodhia R, Saidman G, Schbib V, Peña B, Monteoliva G, Martinez-Castellanos MA, Weeks WB, Lavista Ferres JM. AI-Enabled Screening for Retinopathy of Prematurity in Low-Resource Settings. JAMA Netw Open. 2025 Apr 1;8(4):e257831. doi: 10.1001/jamanetworkopen.2025.7831. PMID: 40299381; PMCID: PMC12042057. E IN GENERALE SOSTENERE TECNOLOGIA E PROGRAMMI MIRATI Al-Khaled T, Valikodath NG, Patel SN, Cole E, Chervinko M, Douglas CE, Tsai ASH, Wu WC, Campbell JP, Chiang MF, Paul Chan RV. Addressing the Third Epidemic of Retinopathy of Prematurity Through Telemedicine and Technology: A Systematic Review. J Pediatr Ophthalmol Strabismus. 2021 Jul-Aug;58(4):261-269. doi: 10.3928/01913913-20210223-01. Epub 2021 Jul 1. PMID: 34288773; PMCID: PMC12285207; Barrero-Castillero A, Corwin BK, VanderVeen DK, Wang JC. Workforce Shortage for Retinopathy of Prematurity Care and Emerging Role of Telehealth and Artificial Intelligence. Pediatr Clin North Am. 2020 Aug;67(4):725-733. doi: 10.1016/j.pcl.2020.04.012. PMID: 32650869.)

DAVE

Javitt J, Dei Cas R, Chiang YP. Cost-effectiveness of screening and cryotherapy for threshold retinopathy of prematurity. Pediatrics. 1993 May;91(5):859-66. PMID: 8474803.

Alder CJ, Mutangana F, Phillips V, Becker ER, Fleming NS, Isenberg SJ, Lambert SR, Frank TD. Cost-Effectiveness of Addressing Retinopathy of Prematurity in Rwanda. Ophthalmic Epidemiol. 2025 Jun;32(3):341-349. doi: 10.1080/09286586.2024.2372803. Epub 2024 Aug 15. PMID: 39146466.

ZIN

Alder CJ, Mutangana F, Phillips V, Becker ER, Fleming NS, Isenberg SJ, Lambert SR, Frank TD. Cost-Effectiveness of Addressing Retinopathy of Prematurity in Rwanda. Ophthalmic Epidemiol. 2025 Jun;32(3):341-349. doi: 10.1080/09286586.2024.2372803. Epub 2024 Aug 15. PMID: 39146466. (SIMILAR)

LAI

Kovács G, Abonyi-Tóth Z, Fadgyas-Freyler P, Kaló Z. Incremental cost of premature birth - a public health care payer perspective from Hungary. BMC Health Serv Res. 2023 Jun 24;23(1):686. doi: 10.1186/s12913-023-09697-w. PMID: 37353814; PMCID: PMC10290341.

Del Busto Wilhelm E, Zimmermann Paiz MA, Ordóñez Rivas AM, Quezada Del Cid NC, Burgos Elías VY, Rousselin Monterroso AA. Cost of screening and timely treatment versus the comprehensive management of the severe visual impairment due to retinopathy of premature. Andes Pediatr. 2023 Jun;94(3):333-338. English, Spanish. doi: 10.32641/andespediatr.v94i3.4530. PMID: 37909936.

Pillay T, Rivero-Arias O, Armstrong N, Seaton SE, Yang M, Banda VL, Dawson K, Ismail AQ, Bountziouka V, Cupit C, Paton A, Manktelow BN, Draper ES, Modi N, Campbell HE, Boyle EM. Optimising neonatal services for very preterm births between 27^+0^ and 31^+6^ weeks gestation in England: the OPTI-PREM mixed-methods study. Health Soc Care Deliv Res. 2025 Apr;13(12):1-126. doi: 10.3310/JYWC6538. PMID: 40232009. (DA USARE COME COMPARAZIONE, TROVATO IN PUBMED COME DISAMBIGUAZIONE)

NAGUIB

Yang B, Lian C, Tian R, Chen Y, Tang S, Xiang H, He H, Zhang G. Twelve-year outcomes of bedside laser photocoagulation for severe retinopathy of prematurity. Front Pediatr. 2023 Jun 23;11:1189236. doi: 10.3389/fped.2023.1189236. PMID: 37425279; PMCID: PMC10327642.

Kamholz KL, Cole CH, Gray JE, Zupancic JA. Cost-effectiveness of early treatment for retinopathy of prematurity. Pediatrics. 2009 Jan;123(1):262-9. doi: 10.1542/peds.2007-2725. PMID: 19117891.

Yu TY, Donovan T, Armfield N, Gole GA. Retinopathy of prematurity: the high cost of screening regional and remote infants. Clin Exp Ophthalmol. 2018 Aug;46(6):645-651. doi: 10.1111/ceo.13160. Epub 2018 Mar 4. PMID: 29369477.

Brown GC, Brown MM, Sharma S, Tasman W, Brown HC. Cost-effectiveness of treatment for threshold retinopathy of prematurity. Pediatrics. 1999 Oct;104(4):e47. doi: 10.1542/peds.104.4.e47. PMID: 10506272.

van den Akker-van Marle ME, van Sorge AJ, Schalij-Delfos NE. Cost and effects of risk factor guided screening strategies for retinopathy of prematurity for different treatment strategies. Acta Ophthalmol. 2015 Dec;93(8):706-12. doi: 10.1111/aos.12798. Epub 2015 Jul 6. PMID: 26149829.

Lee SK, Normand C, McMillan D, Ohlsson A, Vincer M, Lyons C; Canadian Neonatal Network. Evidence for changing guidelines for routine screening for retinopathy of prematurity. Arch Pediatr Adolesc Med. 2001 Mar;155(3):387-95. doi: 10.1001/archpedi.155.3.387. PMID: 11231807.

Hariharan L, Gilbert CE, Quinn GE, Barg FK, Lomuto C, Quiroga A, McLeod-Omawale J, Zin A, Ortiz Z, Alda E, Bouzas L, Brussa M, Cattaino A, Dinerstein A, Erpen N, Fandiño A, Galina L, Manzitti J, Marinaro S, Sepúlveda T, Visintín P, Silva JC, Magluta C, Benitez A. Reducing Blindness from Retinopathy of Prematurity (ROP) in Argentina Through Collaboration, Advocacy and Policy Implementation. Health Policy Plan. 2018 Jun 1;33(5):654-665. doi: 10.1093/heapol/czy004. PMID: 29668967.

Castillo-Riquelme MC, Lord J, Moseley MJ, Fielder AR, Haines L. Cost-effectiveness of digital photographic screening for retinopathy of prematurity in the United Kingdom. Int J Technol Assess Health Care. 2004 Spring;20(2):201-13. doi: 10.1017/s0266462304000984. PMID: 15209180.

Yanovitch TL, Siatkowski RM, McCaffree M, Corff KE. Retinopathy of prematurity in infants with birth weight>or=1250 grams-incidence, severity, and screening guideline cost-analysis. J AAPOS. 2006 Apr;10(2):128-34. doi: 10.1016/j.jaapos.2005.08.005. PMID: 16678747.

Wintergerst MWM, Petrak M, Li JQ, Larsen PP, Berger M, Holz FG, Finger RP, Krohne TU. Non-contact smartphone-based fundus imaging compared to conventional fundus imaging: a low-cost alternative for retinopathy of prematurity screening and documentation. Sci Rep. 2019 Dec 23;9(1):19711. doi: 10.1038/s41598-019-56155-x. PMID: 31873142; PMCID: PMC6928229.

Dunbar JA, Hsu V, Christensen M, Black B, Williams P, Beauchamp G. Cost-utility analysis of screening and laser treatment of retinopathy of prematurity. J AAPOS. 2009 Apr;13(2):186-90. doi: 10.1016/j.jaapos.2008.10.014. PMID: 19393519.

Vinekar A, Mangalesh S, Jayadev C, Gilbert C, Dogra M, Shetty B. Impact of expansion of telemedicine screening for retinopathy of prematurity in India. Indian J Ophthalmol. 2017 May;65(5):390-395. doi: 10.4103/ijo.IJO_211_17. PMID: 28573995; PMCID: PMC5565892.

Alda E, Lomuto CC, Benítez AM, Bouzas L, Brussa M, Cattaino A, Dinerstein NA, Erpen N, Galina L, Mansilla C, Marinaro S, Quiroga A, Saidman G, Sánchez C, Sepúlveda T, Visintin P. Results of the National Program for the Prevention of Blindness in Childhood by Retinopathy of Prematurity in Argentina (2004-2016). Arch Argent Pediatr. 2018 Dec 1;116(6):386-393. English, Spanish. doi: 10.5546/aap.2018.eng.386. PMID: 30457716.

Varughese S, Gilbert C, Pieper C, Cook C. Retinopathy of prematurity in South Africa: an assessment of needs, resources and requirements for screening programmes. Br J Ophthalmol. 2008 Jul;92(7):879-82. doi: 10.1136/bjo.2008.137588. PMID: 18577635.

Isaac M, Isaranuwatchai W, Tehrani N. Cost analysis of remote telemedicine screening for retinopathy of prematurity. Can J Ophthalmol. 2018 Apr;53(2):162-167. doi: 10.1016/j.jcjo.2017.08.018. Epub 2017 Nov 14. PMID: 29631829.

Prinja S, Bahuguna P, Mohan P, Mazumder S, Taneja S, Bhandari N, van den Hombergh H, Kumar R. Cost Effectiveness of Implementing Integrated Management of Neonatal and Childhood Illnesses Program in District Faridabad, India. PLoS One. 2016 Jan 4;11(1):e0145043. doi: 10.1371/journal.pone.0145043. PMID: 26727369; PMCID: PMC4699694.

Arnesen L, Durán P, Silva J, Brumana L. A multi-country, cross-sectional observational study of retinopathy of prematurity in Latin America and the Caribbean. Rev Panam Salud Publica. 2016 Jun;39(6):322-329. PMID: 27706429.

Padhi TR, Pradhan L, Padhy SK, Meherda A, Samantaray B, Patro KK, Devi S, Mishra M, Mahapatra S, Sutar S, Nayak SR, Kelgaonkar A, Khalsa A, Shukla R, Gilbert C. Retinopathy of prematurity care in peripheral districts in Odisha, India: Pilot for a sustainable model. Indian J Ophthalmol. 2020 Feb;68(Suppl 1):S124-S127. doi: 10.4103/ijo.IJO_1914_19. PMID: 31937747; PMCID: PMC7001188.

Haines L, Fielder AR, Baker H, Wilkinson AR. UK population based study of severe retinopathy of prematurity: screening, treatment, and outcome. Arch Dis Child Fetal Neonatal Ed. 2005 May;90(3):F240-4. doi: 10.1136/adc.2004.057570. PMID: 15846016; PMCID: PMC1721880.

Goyal A, Giridhar A, Gopalakrishnan M, Thachil T. Neonatal Intensive Care Unit-based screening program for retinopathy of prematurity and its treatment in an Indian population. Indian J Ophthalmol. 2019 Jun;67(6):828-833. doi: 10.4103/ijo.IJO_201_18. PMID: 31124496; PMCID: PMC6552612.

Afarid M, Hosseini H, Abtahi B. Screening for retinopathy of prematurity in South of Iran. Middle East Afr J Ophthalmol. 2012 Jul-Sep;19(3):277-81. doi: 10.4103/0974-9233.97922. PMID: 22837619; PMCID: PMC3401795.

(ALDER – ROTHSCHILD – ZIN -

MOITRY

Vinekar A, Jayadev C, Mangalesh S, Shetty B, Vidyasagar D. Role of tele-medicine in retinopathy of prematurity screening in rural outreach centers in India - a report of 20,214 imaging sessions in the KIDROP program. Semin Fetal Neonatal Med. 2015 Oct;20(5):335-45. doi: 10.1016/j.siny.2015.05.002. Epub 2015 Jun 17. PMID: 26092301.

Vinekar A, Avadhani K, Dogra M, Sharma P, Gilbert C, Braganza S, Shetty B. A novel, low-cost method of enrolling infants at risk for Retinopathy of Prematurity in centers with no screening program: the REDROP study. Ophthalmic Epidemiol. 2012 Oct;19(5):317-21. doi: 10.3109/09286586.2012.698358. Epub 2012 Aug 16. PMID: 22897645.

Mgharbil E, Raffa LH, Alessa S, Alamri A. Screening premature infants for retinopathy of prematurity in a tertiary hospital in Saudi Arabia. Ann Saudi Med. 2020 Mar-Apr;40(2):87-93. doi: 10.5144/0256-4947.2020.87. Epub 2020 Apr 2. PMID: 32241163; PMCID: PMC7118231.

Mataftsi A, Moutzouri S, Karagianni P, Ziakas N, Soubasi V, Brazitikos P, Haidich AB. Retinopathy of prematurity occurrence and evaluation of screening policy in a large tertiary Greek cohort. Int Ophthalmol. 2020 Feb;40(2):385-391. doi: 10.1007/s10792-019-01195-5. Epub 2019 Oct 22. PMID: 31641909.

Goyal A, Giridhar A, Gopalakrishnan M, Thachil T. Neonatal Intensive Care Unit-based screening program for retinopathy of prematurity and its treatment in an Indian population. Indian J Ophthalmol. 2019 Jun;67(6):828-833. doi: 10.4103/ijo.IJO_201_18. PMID: 31124496; PMCID: PMC6552612.

Azad R, Chandra P, Gangwe A, Kumar V. Lack of Screening Underlies Most Stage-5 Retinopathy of Prematurity among Cases Presenting to a Tertiary Eye Center in India. Indian Pediatr. 2016 Nov 7;53 Suppl 2:S103-S106. PMID: 27915316.

Maka E, Kovács G, Imre L, Gilbert C, Szabó M, Németh J, Nagy ZZ, Somogyvári Z. The validity of telemedicine-based screening for retinopathy of prematurity in the Premature Eye Rescue Program in Hungary. J Telemed Telecare. 2021 Jul;27(6):367-375. doi: 10.1177/1357633X19880113. Epub 2019 Oct 23. PMID: 31645172.

Begley BA, Martin J, Tufty GT, Suh DW. Evaluation of a Remote Telemedicine Screening System for Severe Retinopathy of Prematurity. J Pediatr Ophthalmol Strabismus. 2019 May 22;56(3):157-161. doi: 10.3928/01913913-20190215-01. PMID: 31116862.

Patel SN, Martinez-Castellanos MA, Berrones-Medina D, Swan R, Ryan MC, Jonas KE, Ostmo S, Campbell JP, Chiang MF, Chan RVP; GEN-ROP; i-ROP Research Consortium. Assessment of a Tele-education System to Enhance Retinopathy of Prematurity Training by International Ophthalmologists-in-Training in Mexico. Ophthalmology. 2017 Jul;124(7):953-961. doi: 10.1016/j.ophtha.2017.02.014. Epub 2017 Apr 3. PMID: 28385303; PMCID: PMC7895299.

Chen Y, Feng J, Li F, Yin H, Liang J, Li X. ANALYSIS OF CHANGES IN CHARACTERISTICS OF SEVERE RETINOPATHY OF PREMATURITY PATIENTS AFTER SCREENING GUIDELINES WERE ISSUED IN CHINA. Retina. 2015 Aug;35(8):1674-9. doi: 10.1097/IAE.0000000000000512. PMID: 25719989.

Fleck BW, Wright E, Dhillon B, Millar GT, Laing IA. An audit of the 1995 Royal College of Ophthalmologists guidelines for screening for retinopathy of prematurity applied retrospectively in one regional neonatal intensive care unit. Eye (Lond). 1995;9 ( Pt 6 Su):31-5. PMID: 8729016.

Fielder AR, Haines L, Scrivener R, Wilkinson AR, Pollock JI; Royal Colleges of Ophthalmologists and Paediatrics and Child Health, and the British Association of Perinatal Medicine. Retinopathy of prematurity in the UK II: audit of national guidelines for screening and treatment. Eye (Lond). 2002 May;16(3):285-91. doi: 10.1038/sj.eye.6700131. PMID: 12032719.

Haines L, Fielder AR, Scrivener R, Wilkinson AR; Royal College of Paediatrics and Child Health, the Royal College of Ophthalmologists and British Association of Perinatal Medicine. Retinopathy of prematurity in the UK I: the organisation of services for screening and treatment. Eye (Lond). 2002 Jan;16(1):33-8. doi: 10.1038/sj.eye.6700030. PMID: 11913885.

Wang SK, Callaway NF, Wallenstein MB, Henderson MT, Leng T, Moshfeghi DM. SUNDROP: six years of screening for retinopathy of prematurity with telemedicine. Can J Ophthalmol. 2015 Apr;50(2):101-6. doi: 10.1016/j.jcjo.2014.11.005. PMID: 25863848.

Fijalkowski N, Zheng LL, Henderson MT, Wang SK, Wallenstein MB, Leng T, Moshfeghi DM. Stanford University Network for Diagnosis of Retinopathy of Prematurity (SUNDROP): five years of screening with telemedicine. Ophthalmic Surg Lasers Imaging Retina. 2014 Mar-Apr;45(2):106-13. doi: 10.3928/23258160-20140122-01. Epub 2014 Jan 20. PMID: 24444469.

Fijalkowski N, Zheng LL, Henderson MT, Wallenstein MB, Leng T, Moshfeghi DM. Stanford University Network for Diagnosis of Retinopathy of Prematurity (SUNDROP): four-years of screening with telemedicine. Curr Eye Res. 2013 Feb;38(2):283-91. doi: 10.3109/02713683.2012.754902. Epub 2013 Jan 18. PMID: 23330739.

Desurmont MG, Bremond-Gignac D, Torchin H, Vacherot B, Jarreau PH, Daruich A. Retinopathy of prematurity detection: a retrospective quality improvement project before-after implementation of retinal digital imaging for screening. Eur J Pediatr. 2023 Jul;182(7):3093-3099. doi: 10.1007/s00431-023-04951-z. Epub 2023 Apr 19. PMID: 37076746.

Gilbert C, Shukla R, Murthy GVS, Santosha BVM, Gudlavalleti AG, Mukpalkar S, Yamarthi P, Pendyala S, Puppala A, Edla S, Batchu T; India ROP Partners Implementation Consortium. Retinopathy of prematurity: Overview and highlights of an initiative to integrate prevention, screening, and management into the public health system in India. Indian J Ophthalmol. 2020 Feb;68(Suppl 1):S103-S107. doi: 10.4103/ijo.IJO_2080_19. PMID: 31937743; PMCID: PMC7001169.

Larsen PP, Bründer MC, Petrak M, Jehle V, Lagrèze WA, Holz FG, Stahl A, Krohne TU. Frühgeborenenretinopathie-Screening: Trends über die vergangenen 5 Jahre an zwei deutschen Universitätskliniken [Screening for retinopathy of prematurity: Trends over the past 5 years in two German university hospitals]. Ophthalmologe. 2018 Jun;115(6):469-475. German. doi: 10.1007/s00347-018-0675-3. PMID: 29500715.

Kleberg A, Warren I, Norman E, Mörelius E, Berg AC, Mat-Ali E, Holm K, Fielder A, Nelson N, Hellström-Westas L. Lower stress responses after Newborn Individualized Developmental Care and Assessment Program care during eye screening examinations for retinopathy of prematurity: a randomized study. Pediatrics. 2008 May;121(5):e1267-78. doi: 10.1542/peds.2006-2510. PMID: 18450869.

Barry GP, Tauber K, Emmanuel G, Horgan MJ, Simon JW. The effectiveness of policy changes designed to increase the attendance rate for outpatient retinopathy of prematurity (ROP) screening examinations. J AAPOS. 2013 Jun;17(3):296-300. doi: 10.1016/j.jaapos.2013.03.010. PMID: 23791413.

Quinn GE, Ying GS, Bell EF, Donohue PK, Morrison D, Tomlinson LA, Binenbaum G; G-ROP Study Group. Incidence and Early Course of Retinopathy of Prematurity: Secondary Analysis of the Postnatal Growth and Retinopathy of Prematurity (G-ROP) Study. JAMA Ophthalmol. 2018 Dec 1;136(12):1383-1389. doi: 10.1001/jamaophthalmol.2018.4290. PMID: 30326046; PMCID: PMC6583045.

Attar MA, Gates MR, Iatrow AM, Lang SW, Bratton SL. Barriers to screening infants for retinopathy of prematurity after discharge or transfer from a neonatal intensive care unit. J Perinatol. 2005 Jan;25(1):36-40. doi: 10.1038/sj.jp.7211203. PMID: 15496873.

Jalali S, Anand R, Rani PK, Balakrishnan D. Impact of the day-30 screening strategy on the disease presentation and outcome of retinopathy of prematurity. The Indian twin cities retinopathy of prematurity report number 3. Indian J Ophthalmol. 2014 May;62(5):610-4. doi: 10.4103/0301-4738.118449. PMID: 24088643; PMCID: PMC4065515.

Bulut O, Tarak Bozkurt O, Arslanoglu S. Oral Ibuprofen Versus Oral Paracetamol in Pain Management During Screening for Retinopathy of Prematurity: A Prospective Observational Study. J Perinat Neonatal Nurs. 2022 Jul-Sep 01;36(3):305-311. doi: 10.1097/JPN.0000000000000675. PMID: 35894729.

Marsh VA, Young WO, Dunaway KK, Kissling GE, Carlos RQ, Jones SM, Shockley DH, Weaver NL, Ransom JL, Gal P. Efficacy of topical anesthetics to reduce pain in premature infants during eye examinations for retinopathy of prematurity. Ann Pharmacother. 2005 May;39(5):829-33. doi: 10.1345/aph.1E476. Epub 2005 Mar 29. PMID: 15797982.

Chan H, Cougnard-Grégoire A, Korobelnik JF, Delyfer MN, Touboul D, Coste V, Sarlangue J, Dutheil C, Paya C. Screening for retinopathy of prematurity by telemedicine in a tertiary level neonatal intensive care unit in France: Review of a six-year period. J Fr Ophtalmol. 2018 Dec;41(10):926-932. doi: 10.1016/j.jfo.2018.02.020. Epub 2018 Nov 12. PMID: 30442486.

Binenbaum G, Ying GS, Tomlinson LA; Postnatal Growth and Retinopathy of Prematurity (G-ROP) Study Group. Validation of the Children's Hospital of Philadelphia Retinopathy of Prematurity (CHOP ROP) Model. JAMA Ophthalmol. 2017 Aug 1;135(8):871-877. doi: 10.1001/jamaophthalmol.2017.2295. PMID: 28715553; PMCID: PMC5710287.

Lee JW, SilfaMazara F, Olivier A, McCabe F, Rivera L, Martin RN, Davis JM. Retinopathy of prematurity in the Dominican Republic: challenges to screening and prevention. Am J Perinatol. 2012 Nov;29(10):801-6. doi: 10.1055/s-0032-1316441. Epub 2012 Jul 6. PMID: 22773287.

Ademola-Popoola DS, Onakoya AO, Ezisi CN, Okeigbemen VW, Aghaji AE, Musa KO, Adio AO, Ibrahim YA, Oguego NC. Case series of retinopathy of prematurity blindness in Nigeria: A wakeup call to policy makers, hospitals, ophthalmologists and paediatricians. Niger Postgrad Med J. 2021 Oct-Dec;28(4):303-306. doi: 10.4103/npmj.npmj_595_21. PMID: 34850760.

Kim E, Nguyen KH, Donovan T, Edirippulige S, Armfield NR. A cost-minimisation analysis comparing alternative telemedicine screening approaches for retinopathy of prematurity. J Telemed Telecare. 2023 Apr;29(3):196-202. doi: 10.1177/1357633X20976028. Epub 2021 Jan 7. PMID: 33412992.

Asturias AL, Gilbert C, Silva JC, Quinn GE. Implementation of telemedicine screening for retinopathy of prematurity in rural areas in Guatemala. J AAPOS. 2022 Feb;26(1):22.e1-22.e5. doi: 10.1016/j.jaapos.2021.08.307. Epub 2021 Dec 29. PMID: 34973448.

Vinekar A, Jayadev C, Mangalesh S, Kurian M, Dogra M, Bauer N, Shetty B. Initiating Retinopathy of Prematurity Screening before Discharge from the Neonatal Care Unit: Effect on Enrolment in Rural India. Indian Pediatr. 2016 Nov 7;53 Suppl 2:S107-S111. PMID: 27915317.

Weaver DT, Murdock TJ. Telemedicine detection of type 1 ROP in a distant neonatal intensive care unit. J AAPOS. 2012 Jun;16(3):229-33. doi: 10.1016/j.jaapos.2012.01.007. PMID: 22681938.

Mehta P, Srivastava S, Aggrohiya D, Garg A. Quality Improvement Initiative to Improve the Screening Rate of Retinopathy of Prematurity in Outborn Neonatal Intensive Care Graduates. Indian Pediatr. 2018 Sep 15;55(9):780-783. PMID: 30345985.

Tram JS, Golding BM, Lim C, Kuschel CA, Elder JE. Changed ophthalmic workload following introduction of digital retinal photography for retinopathy of prematurity screening. Clin Exp Ophthalmol. 2021 May;49(4):368-372. doi: 10.1111/ceo.13926. Epub 2021 Apr 29. PMID: 33788997.

Photographic Screening for Retinopathy of Prematurity (Photo-ROP) Cooperative Group. The photographic screening for retinopathy of prematurity study (photo-ROP). Primary outcomes. Retina. 2008 Mar;28(3 Suppl):S47-54. doi: 10.1097/IAE.0b013e31815e987f. Erratum in: Retina. 2009 Jan;29(1):127. PMID: 18317345.

van Sorge AJ, Termote JU, Simonsz HJ, Kerkhoff FT, van Rijn LJ, Lemmens WA, Schalij-Delfos NE. Outcome and quality of screening in a nationwide survey on retinopathy of prematurity in The Netherlands. Br J Ophthalmol. 2014 Aug;98(8):1056-60. doi: 10.1136/bjophthalmol-2013-304493. Epub 2014 Mar 28. PMID: 24682181.

Yang Q, Zhou X, Ni Y, Shan H, Shi W, Yin X, Liu J, Huang X. Optimised retinopathy of prematurity screening guideline in China based on a 5-year cohort study. Br J Ophthalmol. 2021 Jun;105(6):819-823. doi: 10.1136/bjophthalmol-2020-316401. Epub 2020 Jul 16. PMID: 32675062.

KELKAR

Norman M, Hellström A, Hallberg B, Wallin A, Gustafson P, Tornqvist K, Håkansson S, Holmström G. Prevalence of Severe Visual Disability Among Preterm Children With Retinopathy of Prematurity and Association With Adherence to Best Practice Guidelines. JAMA Netw Open. 2019 Jan 4;2(1):e186801. doi: 10.1001/jamanetworkopen.2018.6801. PMID: 30646195; PMCID: PMC6324320.

Darlow BA, Vento M, Beltempo M, Lehtonen L, Håkansson S, Reichman B, Helenius K, Sjörs G, Sigali E, Lee S, Noguchi A, Morisaki N, Kusuda S, Bassler D, San Feliciano L, Adams M, Isayama T, Shah PS, Lui K; on behalf of the International Network for Evaluating Outcomes (iNeo) of Neonates. Variations in Oxygen Saturation Targeting, and Retinopathy of Prematurity Screening and Treatment Criteria in Neonatal Intensive Care Units: An International Survey. Neonatology. 2018;114(4):323-331. doi: 10.1159/000490372. Epub 2018 Aug 8. PMID: 30089298.

Chow LC, Wright KW, Sola A; CSMC Oxygen Administration Study Group. Can changes in clinical practice decrease the incidence of severe retinopathy of prematurity in very low birth weight infants? Pediatrics. 2003 Feb;111(2):339-45. doi: 10.1542/peds.111.2.339. PMID: 12563061.

Gunn DJ, Cartwright DW, Yuen SA, Gole GA. Treatment of retinopathy of prematurity in extremely premature infants over an 18-year period. Clin Exp Ophthalmol. 2013 Mar;41(2):159-66. doi: 10.1111/j.1442-9071.2012.02839.x. PMID: 22712637.

Gopal DP, Rani PK, Rao HL, Jalali S. Prospective study of factors influencing timely versus delayed presentation of preterm babies for retinopathy of prematurity screening at a tertiary eye hospital in India The Indian Twin Cities ROP Screening (ITCROPS) data base report number 6. Indian J Ophthalmol. 2019 Jun;67(6):855-859. doi: 10.4103/ijo.IJO_561_18. PMID: 31124502; PMCID: PMC6552634.

Gharaibeh A, Khassawneh M, Khriesat W, Alkhatib S, Migdadi Y. Adopting Western Retinopathy of Prematurity Screening Programs in Eastern Countries, are we Screening Properly? Middle East Afr J Ophthalmol. 2011 Jul;18(3):209-13. doi: 10.4103/0974-9233.84047. PMID: 21887075; PMCID: PMC3162732.

Ali E, Al-Shafouri N, Hussain A, Baier RJ. Assessment of WINROP algorithm as screening tool for preterm infants in Manitoba to detect retinopathy of prematurity. Paediatr Child Health. 2017 Jul;22(4):203-206. doi: 10.1093/pch/pxx053. Epub 2017 May 8. PMID: 29479215; PMCID: PMC5804560.

Gunn DJ, Cartwright DW, Gole GA. Prevalence and outcomes of laser treatment of aggressive posterior retinopathy of prematurity. Clin Exp Ophthalmol. 2014 Jul;42(5):459-65. doi: 10.1111/ceo.12280. Epub 2014 Jan 23. PMID: 24330069.

Zepeda-Romero LC, Gilbert C. Limitations in ROP Programs in 32 Neonatal Intensive Care Units in Five States in Mexico. Biomed Res Int. 2015;2015:712624. doi: 10.1155/2015/712624. Epub 2015 Jun 17. PMID: 26167494; PMCID: PMC4488079.

Nkanga ED, Agweye CT, Etim BA, Ochigbo SO, Udoh ME, Nkanga DG, Udoh JJ, Asam-Utiin KO. Retinopathy of Prematurity in the University of Calabar Teaching Hospital, Calabar, Nigeria: An Early Report of a Screening Service. J West Afr Coll Surg. 2021 Jul-Sep;11(3):6-12. doi: 10.4103/jwas.jwas_62_21. Epub 2022 Jul 22. PMID: 36132975; PMCID: PMC9484501.

Li Q, Wang Z, Wang R, Tang H, Chen H, Feng Z. A Prospective Study of the Incidence of Retinopathy of Prematurity in China: Evaluation of Different Screening Criteria. J Ophthalmol. 2016;2016:5918736. doi: 10.1155/2016/5918736. Epub 2016 Jun 13. PMID: 27382487; PMCID: PMC4921623.

Nassar MM. Screening for retinopathy of prematurity: a report from upper Egypt. Int J Ophthalmol. 2016 Feb 18;9(2):262-5. doi: 10.18240/ijo.2016.02.15. PMID: 26949647; PMCID: PMC4761739. LA MANCANZA DI UN PROTOCOLLO!

Dabir S, Mohankumar A, Munusamy S, Rajan M, Azad R. Review of incidental ocular pathologies detected during the screening of retinopathy of prematurity in preterm infants. Oman J Ophthalmol. 2023 Jun 27;16(2):258-262. doi: 10.4103/ojo.ojo_232_22. PMID: 37602168; PMCID: PMC10433055.

Albalawi HB, Hashem F, Alharbi ANJ, Alali NM, Alshehri WMS, Alharfy AAN, Alzahrani AMM, Albalawi NMA, Magliyah MS, Alenezi SH. Retinopathy of Prematurity as Multidisciplinary Approach, a Pediatricians Standpoint, and Practice. J Multidiscip Healthc. 2022 Jan 20;15:153-159. doi: 10.2147/JMDH.S351878. PMID: 35082499; PMCID: PMC8786345.

Zhang RH, Liu YM, Dong L, Li HY, Li YF, Zhou WD, Wu HT, Wang YX, Wei WB. Prevalence, Years Lived With Disability, and Time Trends for 16 Causes of Blindness and Vision Impairment: Findings Highlight Retinopathy of Prematurity. Front Pediatr. 2022 Mar 11;10:735335. doi: 10.3389/fped.2022.735335. PMID: 35359888; PMCID: PMC8962664.

AlBathi L, Abouammoh N, AlSwaina N, AlBalawi HB, Al Qahtani AA, Talea M, AlSulaiman SM, Abouammoh MA. Pitfalls of Advanced Retinopathy of Prematurity Presentation: A Content Analysis of Medical Records. Risk Manag Healthc Policy. 2021 Sep 16;14:3873-3882. doi: 10.2147/RMHP.S326757. PMID: 34557046; PMCID: PMC8453427.

Choudhary RA, Hashmi S, Tayyab H. Smartphone-based fundus imaging for evaluation of Retinopathy of Prematurity in a low-income country: A pilot study. Pak J Med Sci. 2023 May-Jun;39(3):638-643. doi: 10.12669/pjms.39.3.7053. PMID: 37250571; PMCID: PMC10214799.

Bain LC, Kristensen-Cabrera AI, Lee HC. A Qualitative Analysis of Challenges and Successes in Retinopathy of Prematurity Screening. AJP Rep. 2018 Apr;8(2):e128-e133. doi: 10.1055/s-0038-1660519. Epub 2018 Jun 11. PMID: 29896443; PMCID: PMC5995725.

Smith AF, Sadeq A, Kinzel E, Bhambhwani V. A Systematic Review of Economic Evaluations Conducted for Interventions to Screen, Treat, and Manage Retinopathy of Prematurity (ROP) in the United States, United Kingdom, and Canada. Ophthalmic Epidemiol. 2023 Apr;30(2):113-120. doi: 10.1080/09286586.2022.2084757. Epub 2022 Jun 14. PMID: 35698819.

Ndukwe T, Cole E, Scanzera AC, Chervinko MA, Chiang MF, Campbell JP, Chan RVP. Health Equity and Disparities in ROP Care: A Need for Systematic Evaluation. Front Pediatr. 2022 Apr 1;10:806691. doi: 10.3389/fped.2022.806691. PMID: 35433564; PMCID: PMC9010777.

Raj S, Kumar P. Development and validation of education materials to reduce childhood blindness due to retinopathy of prematurity. J Educ Health Promot. 2021 Sep 30;10:342. doi: 10.4103/jehp.jehp_687_20. PMID: 34761028; PMCID: PMC8552265.

Al Amro SA, Al Ghamdi SM, Abouammoh MA, Al Aql F, Ahmad K, Alsulaiman SM. Saudi Arabian retinopathy of prematurity national telemedicine program: Achievements and challenges. Saudi J Ophthalmol. 2022 Oct 14;36(3):278-282. doi: 10.4103/sjopt.sjopt_93_22. PMID: 36276246; PMCID: PMC9583349.

Padhi TR, Bhunia S, Das T, Nayak S, Jalan M, Rath S, Barik B, Ali H, Rani PK, Routray D, Jalali S. Outcome of real-time telescreening for retinopathy of prematurity using videoconferencing in a community setting in Eastern India. Indian J Ophthalmol. 2024 May 1;72(5):697-703. doi: 10.4103/IJO.IJO_2024_23. Epub 2024 Feb 23. PMID: 38389241; PMCID: PMC11168531.

Puri S, Sarpal S, Ashat M. Screening of retinopathy of prematurity: a neglected public health issue. Ann Med Health Sci Res. 2014 Mar;4(Suppl 1):S65-6. doi: 10.4103/2141-9248.131726. PMID: 25031916; PMCID: PMC4083728.

Katoch D, Dogra M. The role of advocacy and communication in reducing ROP in India. Community Eye Health. 2018;31(101):S32-S34. PMID: 30275669; PMCID: PMC6157802.

Goyal A, Gopalakrishnan M, Anantharaman G, Chandrashekharan DP, Thachil T, Sharma A. Smartphone guided wide-field imaging for retinopathy of prematurity in neonatal intensive care unit - a Smart ROP (SROP) initiative. Indian J Ophthalmol. 2019 Jun;67(6):840-845. doi: 10.4103/ijo.IJO_1177_18. PMID: 31124499; PMCID: PMC6552601.

KANA

van der Lecq T, Rhoda N, Jordaan E, Seobi T, Visser L, Gilbert C, Muloiwa R, Holmström G; ROPSA Collaborative Group. Screening for retinopathy of prematurity (ROP) in South Africa: data from a newly established prospective regional register. BMJ Open Ophthalmol. 2025 Apr 7;10(1):e002036. doi: 10.1136/bmjophth-2024-002036. PMID: 40194866; PMCID: PMC11979502.

>>>> Ortiz-Seller A, Martorell P, Barranco H, Pascual-Camps I, Morcillo E, Ortiz JL. Comparison of different agents and doses of anti-vascular endothelial growth factors (aflibercept, bevacizumab, conbercept, ranibizumab) versus laser for retinopathy of prematurity: A network meta-analysis. Surv Ophthalmol. 2024 Jul-Aug;69(4):585-605. doi: 10.1016/j.survophthal.2024.02.005. Epub 2024 Mar 1. PMID: 38432359. >>>>> Chen J, Hao Q, Zhang J, Du Y, Chen H, Cheng X. The efficacy and ocular safety following aflibercept, conbercept, ranibizumab, bevacizumab, and laser for retinopathy of prematurity: a systematic review and meta-analysis. Ital J Pediatr. 2023 Oct 9;49(1):136. doi: 10.1186/s13052-023-01543-3. PMID: 37814332; PMCID: PMC10561404. >>>> Linghu D, Cheng Y, Zhu X, Deng X, Yin H, Jiang Y, Zhao M, Li X, Liang J. Comparison of Intravitreal Anti-VEGF Agents With Laser Photocoagulation for Retinopathy of Prematurity of 1,627 Eyes in China. Front Med (Lausanne). 2022 May 31;9:911095. doi: 10.3389/fmed.2022.911095. PMID: 35712119; PMCID: PMC9193577.

KELKAR 2

Darlow BA, Elder MJ, Horwood LJ, Donoghue DA, Henderson-Smart DJ; Australian and New Zealand Neonatal Network. Does observer bias contribute to variations in the rate of retinopathy of prematurity between centres? Clin Exp Ophthalmol. 2008 Jan-Feb;36(1):43-6. doi: 10.1111/j.1442-9071.2007.01652.x. PMID: 18290953.

Reynolds JD, Dobson V, Quinn GE, Fielder AR, Palmer EA, Saunders RA, Hardy RJ, Phelps DL, Baker JD, Trese MT, Schaffer D, Tung B; CRYO-ROP and LIGHT-ROP Cooperative Study Groups. Evidence-based screening criteria for retinopathy of prematurity: natural history data from the CRYO-ROP and LIGHT-ROP studies. Arch Ophthalmol. 2002 Nov;120(11):1470-6. doi: 10.1001/archopht.120.11.1470. PMID: 12427059.

Pirelli A, Savant Levet P, Garetti E, Ancora G, Merazzi D, Bellieni CV, Lago P; Pain Study Group of the Italian Society of Neonatology. Literature review informs clinical guidelines for pain management during screening and laser photocoagulation for retinopathy of prematurity. Acta Paediatr. 2019 Apr;108(4):593-599. doi: 10.1111/apa.14523. Epub 2018 Aug 29. PMID: 30054933.

ROTHSCHILD

Bhatia SK, Siegel L, Braverman R, Enzenauer R, Granet DB, Robbins SL. Socioeconomics of retinopathy of prematurity screening and treatment in the United States. J AAPOS. 2021 Aug;25(4):227.e1-227.e6. doi: 10.1016/j.jaapos.2021.03.014. Epub 2021 Jul 13. PMID: 34271210.

Joish VN, Malone DC, Miller JM. A cost-benefit analysis of vision screening methods for preschoolers and school-age children. J AAPOS. 2003 Aug;7(4):283-90. doi: 10.1016/s1091-8531(03)00116-2. PMID: 12917617.

GILBERT & TODD

Moin M, Mian LS, Martinez-Hussain M, Shahid M, Gilbert C, Mian UK. Severe ROP rate and assessment of the burden of ROP screening at a single tertiary care public hospital in Pakistan. BMC Ophthalmol. 2025 Oct 23;25(1):594. doi: 10.1186/s12886-025-04437-6. PMID: 41131487; PMCID: PMC12548204.

JFC Mutangana F, Muhizi C, Mudereva G, Noë P, Musiime S, Ngambe T, Tuyisenge L, Kumwami M, Nsabimana H, Conard C, Fonteyne Y, Casteels I, De Smedt S; Rwanda Retinopathy of Prematurity Study Group. Retinopathy of prematurity in Rwanda: a prospective multi-centre study following introduction of screening and treatment services. Eye (Lond). 2020 May;34(5):847-856. doi: 10.1038/s41433-019-0529-5. Epub 2019 Jul 30. Erratum in: Eye (Lond). 2020 May;34(5):992. doi: 10.1038/s41433-019-0561-5. PMID: 31363176; PMCID: PMC7182562.; Janevic T, Zeitlin J, Auger N, Egorova NN, Hebert P, Balbierz A, Howell EA. Association of Race/Ethnicity With Very Preterm Neonatal Morbidities. JAMA Pediatr. 2018 Nov 1;172(11):1061-1069. doi: 10.1001/jamapediatrics.2018.2029. PMID: 30208467; PMCID: PMC6248139.

Ryckman KK, Holdefer PJ, Sileo E, Carlson C, Weathers N, Jasper EA, Cho H, Oltman SP, Dagle JM, Jelliffe-Pawlowski LL, Rogers EE. The validity of hospital diagnostic and procedure codes reflecting morbidity in preterm neonates born <32 weeks gestation. J Perinatol. 2023 Nov;43(11):1374-1378. doi: 10.1038/s41372-023-01685-6. Epub 2023 May 3. PMID: 37138163; PMCID: PMC10860645.

Kavitha V, Heralgi MM. Analysis of regular screening of children in schools for the blind - Its importance! Indian J Ophthalmol. 2021 Sep;69(9):2540. doi: 10.4103/ijo.IJO_1836_21. PMID: 34427265; PMCID: PMC8544072.

Chang KM, Patel DK, Tajunisah I, Subrayan V. The trend of retinopathy of prematurity in Malaysia from 1992 to 2001 based on a nationwide blind schools study. Asia Pac J Public Health. 2015 Mar;27(2):217-24. doi: 10.1177/1010539512455047. Epub 2012 Aug 10. PMID: 22887807.

SNOWBALLING SEARCH

Hungi B, Vinekar A, Datti N, Kariyappa P, Braganza S, Chinnaiah S, Donthi K, Shetty B. Retinopathy of Prematurity in a rural Neonatal Intensive Care Unit in South India--a prospective study. Indian J Pediatr. 2012 Jul;79(7):911-5. doi: 10.1007/s12098-012-0707-y. Epub 2012 Feb 23. PMID: 22359197.

Holmström G, Hellström A, Jakobsson P, Lundgren P, Tornqvist K, Wallin A. Evaluation of new guidelines for ROP screening in Sweden using SWEDROP - a national quality register. Acta Ophthalmol. 2015 May;93(3):265-8. doi: 10.1111/aos.12506. Epub 2014 Jul 18. PMID: 25044161.
